# Altered skin microbiome, inflammation, and JAK/STAT signaling in Southeast Asian ichthyosis patients

**DOI:** 10.1186/s40246-024-00603-x

**Published:** 2024-04-16

**Authors:** Minh Ho, Huynh-Nga Nguyen, Minh Van Hoang, Tien Thuy Thi Bui, Bao-Quoc Vu, Truc Huong Thi Dinh, Hoa Thi My Vo, Diana C. Blaydon, Sherif A. Eldirany, Christopher G. Bunick, Chi-Bao Bui

**Affiliations:** 1 Department of Dermatology and Program in Translational Biomedicine, Yale University, New Haven, CT USA; 2 Microbial Genomics DNA Medical Technology, Ho Chi Minh, Vietnam; 3 Department of Biology, Dalat University, Da Lat, Lam Dong Vietnam; 4 Vietnam Vascular Anomalies Center, University Medical Center 3, Ho Chi Minh, Vietnam; 5 Hung Vuong Maternal’s Hospital, Ho Chi Minh, Vietnam; 6 Department of Pathophysiology and Immunology, Can Tho University of Medicine and Pharmacy, Can Tho, Vietnam; 7 Oxford University Clinical Research Unit, Ho Chi Minh, Vietnam; 8 Department of Microbiology, City Children’s Hospital, Ho Chi Minh, Vietnam; 9 Centre for Cell Biology and Cutaneous Research, Blizard Institute, Queen Mary University of London, London, UK; 10 School of Medicine, Vietnam National University, Ho Chi Minh, Vietnam

**Keywords:** Cytokine signaling, Genetic mutation, Immune system activation and profiling, Stem cell inflammatory memory, Sepsis, Microbial infection and dysbiosis, Wound healing, Dermatologic diseases, Scaling and erythema, Physiologic protein mutations

## Abstract

**Background:**

Congenital ichthyosis (CI) is a collective group of rare hereditary skin disorders. Patients present with epidermal scaling, fissuring, chronic inflammation, and increased susceptibility to infections. Recently, there is increased interest in the skin microbiome; therefore, we hypothesized that CI patients likely exhibit an abnormal profile of epidermal microbes because of their various underlying skin barrier defects. Among recruited individuals of Southeast Asian ethnicity, we performed skin meta-genomics (i.e., whole-exome sequencing to capture the entire multi-kingdom profile, including fungi, protists, archaea, bacteria, and viruses), comparing 36 CI patients (representing seven subtypes) with that of 15 CI age-and gender-matched controls who had no family history of CI.

**Results:**

This case–control study revealed 20 novel and 31 recurrent pathogenic variants. Microbiome meta-analysis showed distinct microbial populations, decreases in commensal microbiota, and higher colonization by pathogenic species associated with CI; these were correlated with increased production of inflammatory cytokines and Th17- and JAK/STAT-signaling pathways in peripheral blood mononuclear cells. In the wounds of CI patients, we identified specific changes in microbiota and alterations in inflammatory pathways, which are likely responsible for impaired wound healing.

**Conclusions:**

Together, this research enhances our understanding of the microbiological, immunological, and molecular properties of CI and should provide critical information for improving therapeutic management of CI patients.

**Supplementary Information:**

The online version contains supplementary material available at 10.1186/s40246-024-00603-x.

## Background

The outer epidermis of human skin represents an extremely important physical barrier against body temperature dysregulation, infection, and water loss. The epidermis encompasses a diverse symbiotic microbiome. The healthy human skin microbiome comprises largely the phyla of *Actinobacteria, Firmicutes, Proteobacteria, and Bacteroidetes* [1]—which work cooperatively with stratum corneum biproducts (e.g., neutral/polar lipids and glycosphingolipids [2])—to prevent cutaneous overgrowth of infectious pathogenic microbes [3, 4]. However, homeostasis can be disrupted by mutations/alterations in the human epidermis [5] or skin barrier-processing proteins [6], which increase potential for infection or manifestation of clinical diseases such as atopic dermatitis (AD) or congenital ichthyosis (CI).

For example, case–control studies of AD have shown that the severity of infection is associated with domination of *Staphylococcus aureus* over *Staphylococcus epidermidis* [7], as well as decreased cutaneous microbiome diversity [8]. This example illustrates how the residential microbiome’s composition can influence skin disease activity. A number of studies evaluating the composition of AD [5, 7, 9–12] and psoriasis [13–15] skin microbiome, as well as proposed therapeutic treatments [16, 17], have been published. However, the epidermal microbiome across the various types of CI remains less well characterized.

The congenital ichthyoses comprise at least 20 types, which are divided into non-syndromic versus syndromic forms; they are characterized by malfunction of barrier physiology, abnormal keratinocyte proteins, lipid biosynthesis, cell adhesion, and/or DNA repair [18]. Underlying these various physiologic malfunctions are distinct genetic variants/mutations in a number of genes related to epidermal function; some gene mutations have X-linked inheritance (e.g. *steroid sulfatase* gene mutations causing X-linked ichthyosis), whereas most have autosomal recessive inheritance (e.g. *transglutaminase-1* gene mutations causing lamellar ichthyosis) [18]. It is logical to hypothesize that CI patients, given the genetic, biochemical, and physiologic diversity underlying their skin barrier defects, may harbor distinct, abnormal microbiome signatures.

CI typically displays scaling of the skin (hence, its name, which originates from the Greek word “ichthys,” i.e., “fish,” to describe a fish-scale-like trait). The severity of CI ranges from dry skin (xerosis cutis) seen in ichthyosis vulgaris (IV) to life-threatening Harlequin ichthyosis (HI). Some forms of CI can be associated with a severe, often fatal, neonatal outcome due to infection overlaid with an immature or abnormal immune system. Similar to AD and psoriasis, a compromised epidermal barrier in CI patients (e.g., chronic scaling and fissuring) may facilitate access of skin microbiota into sub-corneal layers, via scratch-related lesions, leading to recurrent/chronic skin wounds, inflammation, and high risk of cutaneous and/or systemic infections.

Recently, immunological profiling of ichthyosis demonstrated that Th17-signaling is elevated, especially with the involvement of IL-17 for congenital ichthyosiform erythroderma, lamellar ichthyosis, and epidermolytic ichthyosis [19–21]. Guttman-Yassky and Paller showed the immune profile and lipid metabolism of these 3 types of ichthyoses (CIE, LI, EI) are more similar to psoriasis than to AD; these data suggest the potential for psoriasis drugs as viable therapies for ichthyosis patients [20].

Interestingly, the severity of AD is more closely correlated with cytokine expression than with any precise gene mutation [22]. This finding reveals an important knowledge gap: what is the interrelationship between the skin microbiome, immune profile, and disease pathogenesis in CI? Currently, clinical evaluation, diagnosis, and management of CI includes serological testing, histology, bacterial cultures, and incidence of viral and fungal infections. However, the lack of extensive characterization of the skin microbiome associated with CI disorders limits accurate diagnosis and effective treatment.

There are 5 evolutionary types of geographically-distinct groups: African, Asian, Oceanian, Caucasian and Amerindian [23]. Differences in skin physiology have arisen over many thousands of years of isolation [24]. Ethnic differences in skin-barrier function are reported, especially with Asian skin having higher rates of trans-epidermal water loss and skin reactivity, as compared to African or Caucasian skin [25]. Although ethnic differences in AD among these various geographically-isolated groups have been reported [26], to our knowledge no similar study has been carried out among CI of such groups. The present study represents a beginning: we have carried out a case–control metagenomics analysis of the skin of 36 CI patients and appropriate non-CI patients of southeastern Asian ethnicity.

## Results

### Characterization of clinical and genetic variants in CI patients

**Fig. 1:**
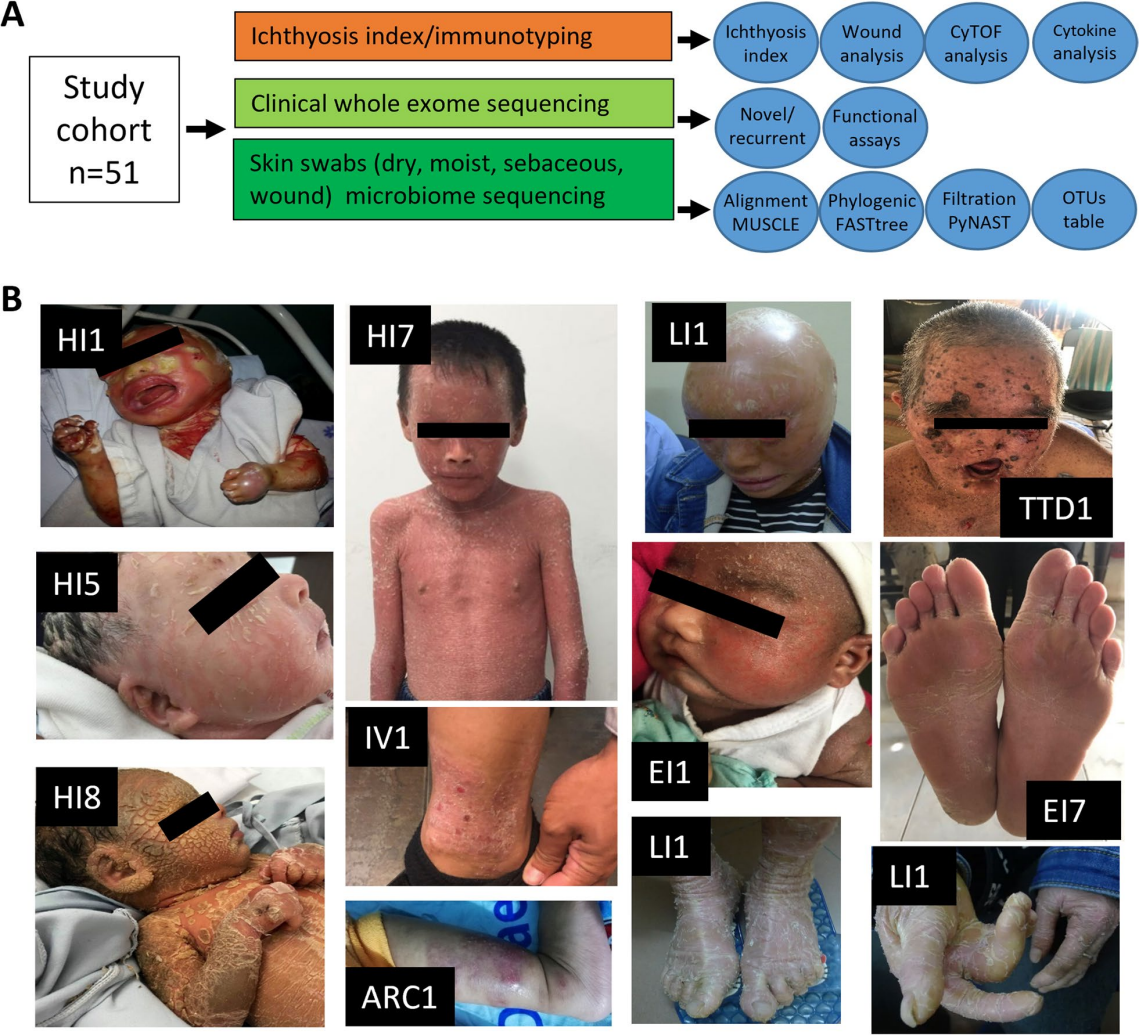
Study design and clinical phenotypes found in Southeast Asian individuals with congenital ichthyosis (CI). **A** Schematic overview of study design and methods used. **B** Clinical photographs of representative individuals with harlequin ichthyosis (HI1, HI5, HI7, HI8), lamellar ichthyosis (LI1), epidermolytic ichthyosis (EI1), trichothiodystrophy/XPD (TTD1), ichthyosis vulgaris (IV1), arthrogryposis, renal dysfunction, and cholestasis (ARC1) syndrome

A study cohort of 51 individuals of Southeast Asian ethnicity (36 CI patients plus 15 age-matched healthy subjects) were assessed clinically and further analyzed by microbiome sequencing, whole exome sequencing (WES), and immunophenotyping (Fig. 1A). First, we examined the clinical patterns of each disorder and compared them to related genetic mutations as defined and categorized by expert dermatologists, microbiologists, and geneticists in Vietnam. We categorized the CI patients (n = 36) into having one of 7 CI disorders: IV (n = 15), HI (n = 8), LI (n = 3), EI (n = 4), Trichothiodystrophy (TTD, n = 3), Arthrogryposis renal dysfunction cholestasis (ARC, n = 1), and Sjögren-Larsson Syndrome (SLS, n = 2) and compared them alongside healthy age-matched subjects (n = 15). The median duration of follow-up was 4.3 years with patient age (average 10.2 years old) ranging from neonatal (birth) to adults (40 years). Clinical phenotypes of some of the CI patients are shown (Fig. 1B).

To characterize the frequency of specific clinical manifestations in CI, our cohort was evaluated for 10 findings: hyperkeratosis, itch, microbial infection, erythroderma, respiratory difficulties, sepsis, collodion membrane at birth, loss of heat, blistering, and cardiac abnormalities. Hyperkeratosis and itch were the most common clinical manifestations of CI (100% and 97.2%, respectively), followed by microbial infection (94.4%) and erythroderma (72.2%) (Table 1). 52.78% of CI patients demonstrated respiratory comorbidities and/or sepsis. There were 6 lethal cases due to infant sepsis (4 HI, 1 LI, and 1 EI). Ten other cases of childhood sepsis were evident in HI (3 out of 8) or in IV (7 out of 15) but were treatable with antibiotics (Additional file 14: Table S1 and Additional file 15: Table S2). Most HI and LI cases also presented with neonatal-onset chronic diarrhea, episodes of fever, recurrent pyodermas, oral candidiasis, and otitis externa. Aside from sepsis, one TTD patient developed necrotizing fasciitis of the nose following *Burkholderia pseudomallei* infection. Interestingly, 19/36 CI patients (53%) exhibited some form of mental health difficulty (Additional file 14: Table S1).

**Table 1:** Clinical phenotypes of congenital ichthyosis (CI) cohort

Clinical manifestations	Patients (# of CI subtype patients with clinical finding) (n = 36)	Frequency (%)
Hyperkeratosis (thick skin)	IV(15), HI(8), LI(3), EI(4), TTD(3), ARC(1), SLS(2)	100
Itch/Pruritus	IV(15), HI(8), LI(3), EI(4), TTD(3), ARC(1), SLS(1)	97.22
Skin microbial infection* (n = 18)	IV(9), HI(2), EI(1), TTD(2), SLS(1)	83.33
Erythroderma	IV(10), HI(8), LI(1), EI(4), TTD(2) SLS(1)	72.22
Respiratory comorbidities	IV(10), HI(3), EI(2), TTD(3), SLS(1)	52.78
Sepsis	IV(7), HI (6), LI(2), EI(1), TTD(3)	52.78
Collodion membrane at birth	IV(1), HI(8), LI(3)	33.33
Cardiomyopathy comorbidities	IV(4), HI (2), EI(2), TTD(3)	30.56
Blistering	HI(8), EI(2)	27.78
Loss of heat	HI(3), LI(1), EI(2)	16.67

The total number of CI patients (n = 36) in our cohort were categorized as: ichthyosis vulgaris (IV, n = 15), harlequin ichthyosis (HI, n = 8), lamellar ichthyosis (LI, n = 3), epidermolytic ichthyosis (EI, n = 4), trichothiodystrophy (TTD, n = 3), arthrogryposis, renal dysfunction, and cholestasis (ARC) syndrome (n = 1), and Sjögren–Larsson syndrome (SLS, n = 2). *Only n = 18 patients involved in testing for this manifestation; data of microbial infection and loss of heat shown in Additional file 15: Tables S2 and Additional file 16: Table S3, respectively

**Fig. 2:**
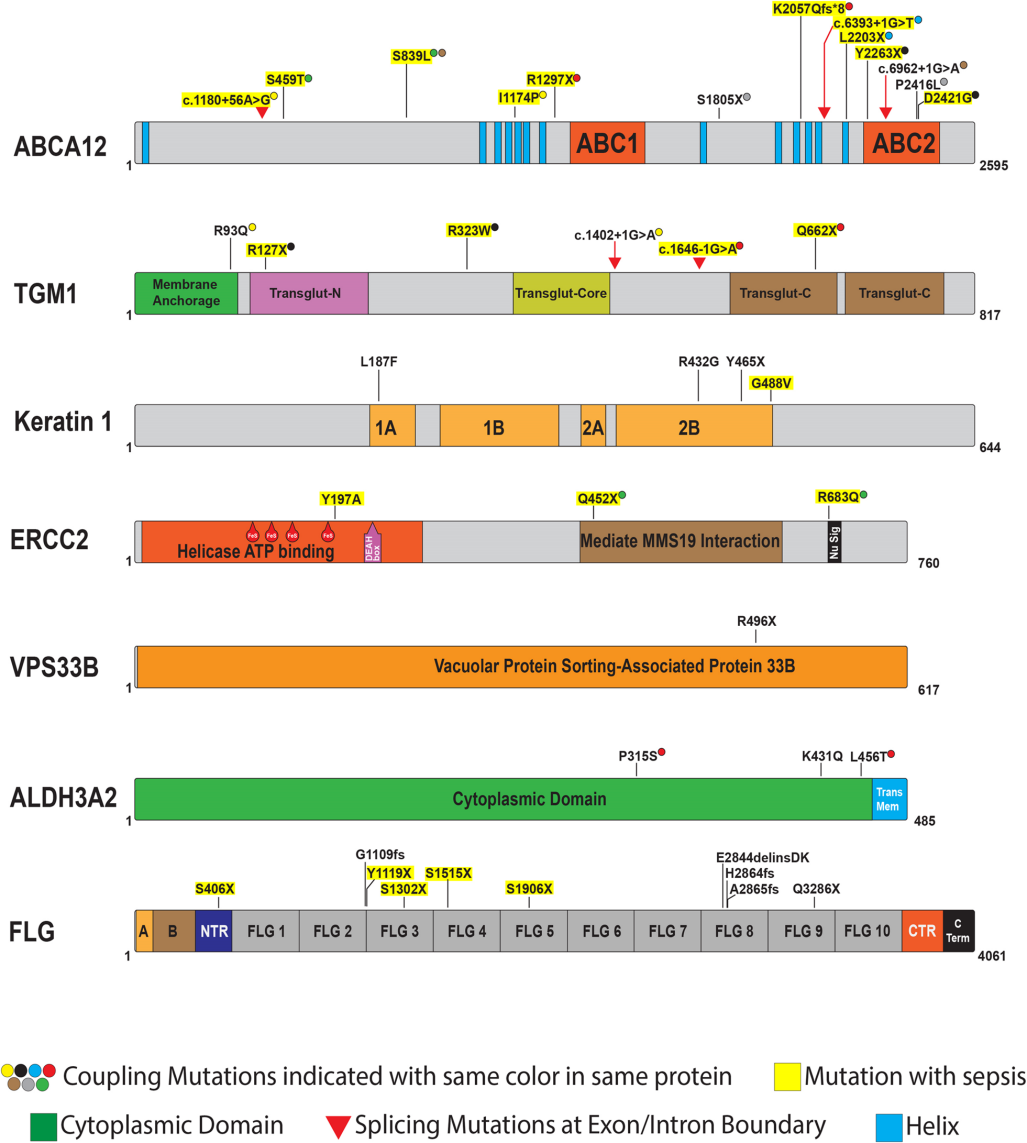
Pathogenic variants identified in cohort of Southeast Asian CI patients. Schematic representation of mutated gene domains with annotated missense, nonsense, splicing, and frameshift mutations identified in affected individuals with *ABCA12, TGM1, KRT1, FLG, ERCC2, VSP33B*, and *ALDH3A2* mutations. “Coupling mutations” refers to variants identified together in the same patient. Variants highlighted with yellow experienced sepsis: we note that most of sepsis cases happened with double mutation, while 70% of the ABCA12 variants associated with sepsis in our cohort were adjacent to the ABC1 or ABC2 domains; KRT1 G488V variant associated with sepsis occurred at the C-terminus in the highly conserved TYR*LLEGE motif known to be critical for intermolecular and higher order filament interactions (see Additional file 3: Fig. S2); IV patients who experienced sepsis had either frameshift or nonsense mutations in the N-terminal half of *FLG* whereas those having mutations more C-terminal did not experience sepsis

Next, we performed WES and genetic linkage analysis in the 36 CI individuals. 31 pathogenic/likely pathogenic mutations (based on American College Medical Genetics) and 20 novel variants were identified (Table 2). Four novel variants were found in more than one CI patient in our cohort. We identified compound heterozygous and homozygous segregated mutations linked to autosomal recessive loss-of-function of *ABCA12, TGM1, ALDH3A2*, and *ERCC2.* The genetic mutations are mapped onto their corresponding protein domain (Fig. 2) and where possible, we analyzed the protein structure alterations caused by the CI-related missense mutations (Additional file 2: Fig. S1, Additional file 3: Fig. S2, Additional file 4: Fig. S3, Additional file 5: Fig. S4).

**Table 2:** Whole exome sequencing (WES) analysis of 36 CI patients

Patient ID	Age at diagnosis	Gene	Variants	Variant classification	Zygosity	Inheritance	Mutation Status
HI1	0–5 years	*ABCA12*: NM_173076.3	R1297X and K2057QfsTer8	Truncating-protein and Truncating-protein	Compound heterozygous	Autosomal recessive	Reported mutation [65] and novel mutation
HI2	0–5 years	*ABCA12*: NM_173076.3	L2203X and c.6393 + 1G > T	Truncating-protein and splicing variant	Compound heterozygous	Autosomal recessive	Novel mutations
HI3	0–5 years	*ABCA12*: NM_173076.3	S459T and S839L	Splicing variant and missense	Compound heterozygous	Autosomal recessive	Reported mutation [66, 67]
HI4	0–5 years	*ABCA12*: NM_173076.3	I1174P and c.1180 + 56A > G	Missense and splicing variant	Compound heterozygous	Autosomal recessive	Novel mutation
HI5	0–5 years	*ABCA12*: NM_173076.3	D2421G and Y2263X	Missense and truncating- protein	Compound heterozygous	Autosomal recessive	Novel mutation
HI6	0–5 years	*ABCA12*: NM_173076.3	D2421G and Y2263X	Missense and truncating-protein	Compound heterozygous	Autosomal recessive	Novel mutation
HI7	0–5 years	*ABCA12*: NM_173076.3	S1805X and P2416L	Truncating-protein and missense	Compound heterozygous	Autosomal recessive	Novel mutation and reported mutation [68]
HI8	0–5 years	*ABCA12*: NM_173076.3	S839L and c.6962 + 1G > A	Missense and splicing variant	Compound heterozygous	Autosomal recessive	Novel mutation
LI1	11–15 years	*TGM1:* NM_000359.3	R127X and R323W	Truncating-protein and missense	Compound heterozygous	Autosomal recessive	Reported mutation [69] and reported mutation [70]
LI2	0–5 years	*TGM1:* NM_000359.3	R93Q and c.1402 + 1G > A	Missense and splicing variant	Compound heterozygous	Autosomal recessive	Reported mutation [71] and novel mutation
LI3	0–5 years	*TGM1:* NM_000359.3	c.1646-1G > A and Q662X	Splicing variant and truncating-protein	Compound heterozygous	Autosomal recessive	Novel mutation and reported mutation [72]
IV1	0–5 years	*FLG:* NM_002016	Q3286X	Truncating-protein	Heterozygous	Autosomal dominant	Novel mutation
IV2	6–10 years	*FLG*: NM_002016	H2864CfsTer5	Truncating-protein	Heterozygous	Autosomal dominant	Reported mutation [73, 74]
IV3	16–20 years	*FLG*: NM_002016	S1906X	Truncating-protein	Heterozygous	Autosomal dominant	Reported mutation [75, 76]
IV4	11–15 years	*FLG*: NM_002016	S1906X	Truncating-protein	Heterozygous	Autosomal dominant	Reported mutation [75, 76]
IV5	6–10 years	*FLG*: NM_002016	H2864CfsTer5	Truncating-protein	Heterozygous	Autosomal dominant	Reported mutation [73, 74]
IV6	6–10 years	*FLG*: NM_002016	A2865GfsTer28	Truncating-protein	Heterozygous	Autosomal dominant	Reported mutation [73, 74]
IV7	16–20 years	*FLG*: NM_002016	E2844delinsDK	Truncating-protein	Heterozygous	Autosomal dominant	Novel mutation
IV8	16–20 years	*FLG*: NM_002016	S1302X	Truncating-protein	Heterozygous	Autosomal dominant	Novel mutation
IV9	61–65 years	*FLG*: NM_002016	S406X	Truncating-protein	Heterozygous	Autosomal dominant	Novel mutation
IV10	16–20 years	*FLG*: NM_002016	S406X	Truncating-protein	Heterozygous	Autosomal dominant	Novel mutation
IV11	26–30 years	*FLG*: NM_002016	S1515X	Truncating-protein	Heterozygous	Autosomal dominant	Reported mutation [77]
IV12	0–5 years	*FLG*: NM_002016	S1515X	Truncating-protein	Heterozygous	Autosomal dominant	Reported mutation [77]
IV13	0–5 years	*FLG*: NM_002016	Y1119X	Truncating-protein	Heterozygous	Autosomal dominant	Novel mutation
IV14	41–45 years	*FLG*: NM_002016	G1109EfsTer13	Truncating-protein	Heterozygous	Autosomal dominant	Reported mutation [78]
IV15	0–5 years	*FLG*: NM_002016	Y1119X	Truncating-protein	Heterozygous	Autosomal dominant	Novel mutation
EI1	0–5 years	*KRT1*: NM_006121.4	G488V	Missense	Heterozygous	Autosomal dominant	Novel mutation
EI2	16–20 years	*KRT1*: NM_006121.4	Y465X	Truncating-protein	Heterozygous	Autosomal dominant	Novel mutation
EI3	6–10 years	*KRT1:* NM_006121.4	R432G	Missense	Heterozygous	Autosomal dominant	Novel mutation
EI4	21–25 years	*KRT1*: NM_006121.4	L187F	Missense	Heterozygous	Autosomal dominant	Reported mutation [79]
TTD1	36–40 years	*ERCC2*: NM_000400	Q452X and R683Q	Truncating-protein and missense	Compound heterozygous	Autosomal recessive	Reported mutation [56]
TTD2	26–30 years	*ERCC2*: NM_000400	Q452X and R683Q	Truncating-protein and missense	Compound heterozygous	Autosomal recessive	Reported mutation [56]
TTD3	26–30 years	*ERCC2*: NM_000400	Y197A	Missense	Homozygous	Autosomal recessive	Novel mutation
ARC1	0–5 years	*VPS33B*: NM_001289148	R496X	Truncating-protein	Homozygous	Autosomal recessive	Reported mutation [80]
SLS1	6–10 years	*ALDH3A2:* NM_001031806.2	P315S and L456T	Missense and missense	Compound heterozygous	Autosomal recessive	Reported mutation [81] and novel mutation
SLS2	6–10 years	*ALDH3A2:* NM_001031806.2	K431Q	Missense	Homozygous	Autosomal recessive	Novel mutation

For each CI patient, age at diagnosis, gene name and sequence identifier, amino acid variant, variant classification, mode of inheritance, and novelty of mutation are presented

To further evaluate our cohort for clinically relevant genotype–phenotype correlations, Fisher exact tests and odds ratio calculations were performed for 12 clinical findings over the 7 distinct CI genotypes (Additional file 18: Table S5). We observed statistically significant association of intensive care unit (ICU) admission with *ABCA12* mutation, death examination with *ABCA12* and *TGM1* mutations, sepsis with *ABCA12* mutation, respiratory problems with *FLG* mutation, collodion membrane at birth with *ABCA12* and *TGM1* mutation, blistering with *ABCA12* and *FLG* mutation, dry and thick skin with *FLG* mutation, impaired wound healing with *FLG* mutation, itch with *ABCA12* and *FLG* mutations, and bacterial infection with *FLG* mutation.

### CI patient microbiome signatures

**Fig. 3:**
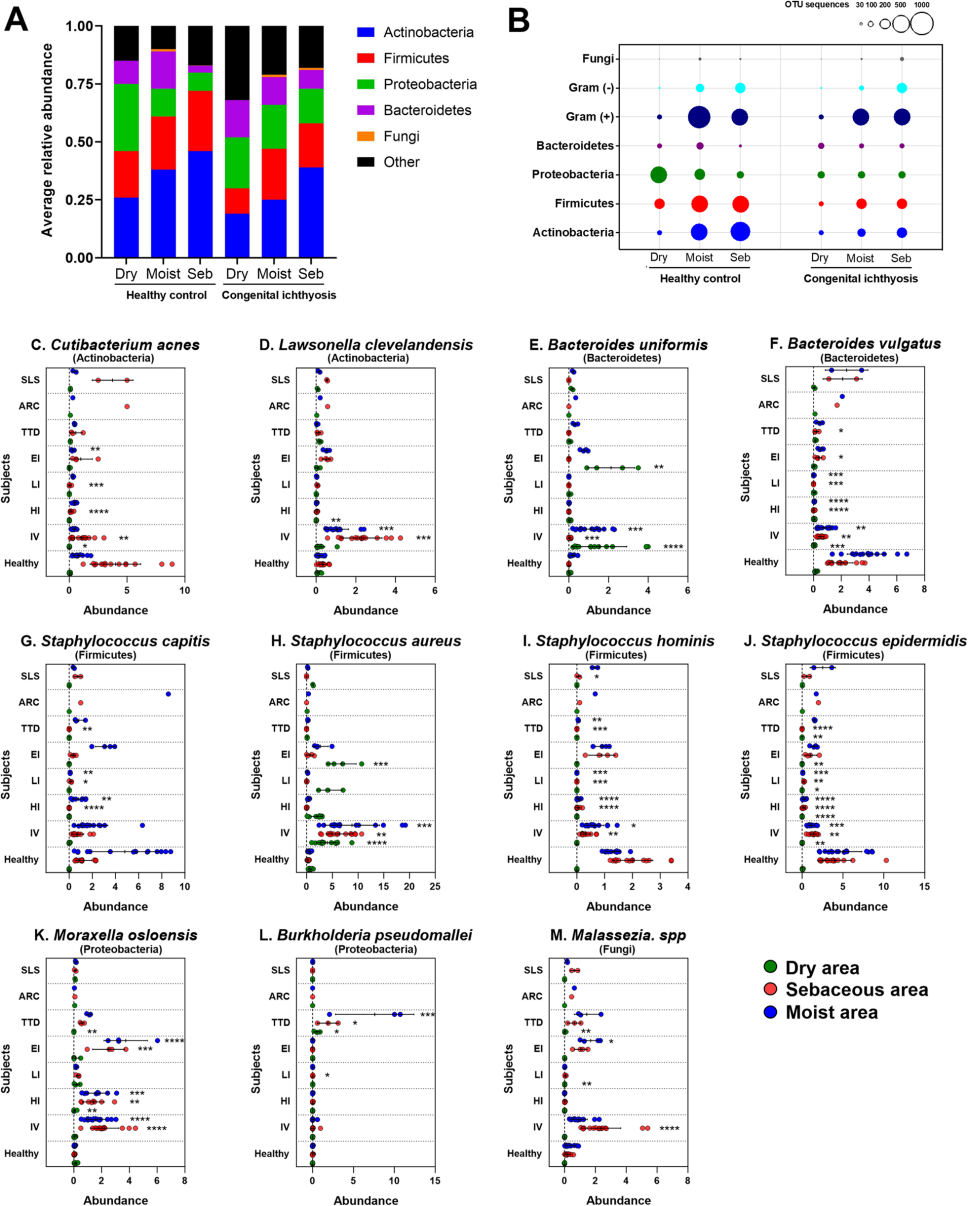
Microbiome profile in CI patients. **A** Bacterial community composition at phylum level (Actinobacteria, Firmicutes, Proteobacteria, Bacteroidetes, fungi, and other) represented as average relative abundance from healthy (left) and CI patients (right) and three different skin sites: dry, moist, and sebaceous. **B** Relative abundance of operational taxonomic units (OTUs) in healthy (left) and CI patients (right) from three different skin sites (dry, moist, and sebaceous) by White’s non-parametric t-test. Circle sizes are proportional to the number of sequences within for the phyla Actinobacteria, Firmicutes, Proteobacteria, Bacteroidetes, fungi, and gram positive/gram negative bacteria. **C**–**M** Abundance of specific bacterial species for each CI clinical type: **C** *Cutibacterium acnes*; **D** *Lawsonella clevelandensis*; E, *Bacteroides uniformis*; **F** *Bacteroides vulgatus*; **G** *Staphylococcus capitis*; **H** *Staphylococcus aureus*; **I** *Staphylococcus hominis*; **J** *Staphylococcus epidermidis;* **K** *Moraxella osloensis*; **L** *Burkholderia pseudomallei*; **M** *Malassezia* species. Data expressed as dots with different colors for dry (green), sebaceous (red), and moist (blue) skin with mean ± SD standard error. *p ≤ 0.05, **p ≤ 0.01, ***p ≤ 0.001, ****p ≤ 0.0001

To better understand the risk of infections in CI, we comprehensively examined the CI patient microbiome (n = 36) by taking superficial swabs of sebaceous skin (facial region), dry skin (olecranal or patella regions) and moist skin (antecubital or popliteal region) and compared the results to age-matched healthy subjects (n = 15; Fig. 1A). We focused on the 4 dominant phyla—Actinobacteria, Firmicutes, Proteobacteria, and Bacteroidetes. Actinobacteria and Firmicutes both showed decreased average relative abundance of colony forming units (cfu) in CI dry, moist, and sebaceous skin compared to the respective locations in healthy controls (Fig. 3A). Proteobacteria showed decreased relative abundance of cfu in dry CI skin compared to healthy controls, but increased abundance in moist and sebaceous skin. Bacteroidetes appeared stable to slightly increased in relative abundance of cfu across dry and sebaceous skin locations in CI patients compared to healthy controls, but slightly decreased in abundance in moist skin. Interestingly, Fungi were increased in sebaceous skin in CI patients compared to healthy controls.

Next, we compared the operational taxonomic units (OTU) sequence reads between the CI patients (n = 36) and healthy controls (n = 15) for dry, moist, and sebaceous skin locations (Fig. 3B). We observed reduced OTU counts of Actinobacteria, Firmicutes, Proteobacteria, and Gram-positive microbes across moist and sebaceous skin, but no significant difference in Bacteroidetes OTU counts (Fig. 3B)*.* Analysis of the OTU sequences at the species level identified a significant reduction in species within the Actinobacteria, Firmicutes, and Bacteroidetes phyla for certain CI types (Fig. 3C–L). For Actinobacteria, CI patients generally had reduced levels of *Cutibacterium acnes* compared to healthy controls*,* with the reduction in *C. acnes* being statistically significant for IV, HI, LI, and EI types (Fig. 3C). *L. clevelandensis* demonstrated a mixed pattern, with significant elevations in IV patients but significant absence in LI and HI patients (Fig. 3D). For the *Bacteroidetes* phylum, *Bacteroides uniformis* was significantly elevated in EI and IV patients, and *B. vulgatus* had significant reductions in IV, HI, LI, EI, and TTD patients (Fig. 3E, F). In the Firmicutes phylum, *Staphylococcus capitis, S. hominis,* and *S. epidermidis* showed significantly reduced levels in all CI patients compared to healthy controls (Fig. 3G, I, J).

In contrast to the reductions in *C. acnes*, *S. capitis*, *S. hominis, S. epidermidis*, and *B. vulgatus*, *S. aureus* of the Firmicutes phylum showed statistically significant elevations in IV and EI patients versus healthy controls, and statistically non-significant elevations in dry skin of LI patients (Fig. 3H). Soil-dwelling and zoonotic disease-associated bacteria from the Proteobacteria phyla were also identified in certain CI patients. *Burkholderia pseudomallei* was exclusively increased in TTD patients (Fig. 3L), whereas *Moraxella osloensis* was increased in multiple CI types, most significantly IV, HI, EI, and TTD (Fig. 3K). Lastly, LI and HI were unique among the CI types in demonstrating a marked reduction in *Malassezia* species, in contrast to IV, EI, and TTD patients who experienced higher *Malassezia* abundance (Fig. 3M).

To evaluate the impact of CI genotype on the presence of various bacteria, we plotted bacterium abundance against CI gene mutation (Additional file 13: Fig. S12). For *C. acnes*, the difference in the amount of *C. acnes* between Healthy (WT) skin and skin with CI variants suggests that genotypes may influence the abundance of *C. acnes*. This may impact biological properties of the skin such as moisture level, pH, lipid barrier, or the skin's immune capabilities determined by the genotypes. *Bacteroides vulgatus* is part of the normal flora of the human skin, but was found in lower abundance in certain CI genotypes. The significant increase in *S. aureus* levels in individuals with *FLG* and *KRT1* genotypes indicate that these genetic variants create favorable conditions for the proliferation of *S. aureus* on the skin. *B. pseudomallei* uniquely was elevated in *ERCC2* genotypes compared to WT and other CI genotypes. Lastly, we observed a significant increase in *Malassezia spp*. between individuals with several genotypes versus those in the WT group.

### Principal co-ordinate analysis identifies CI microbiome subgroups

**Fig. 4:**
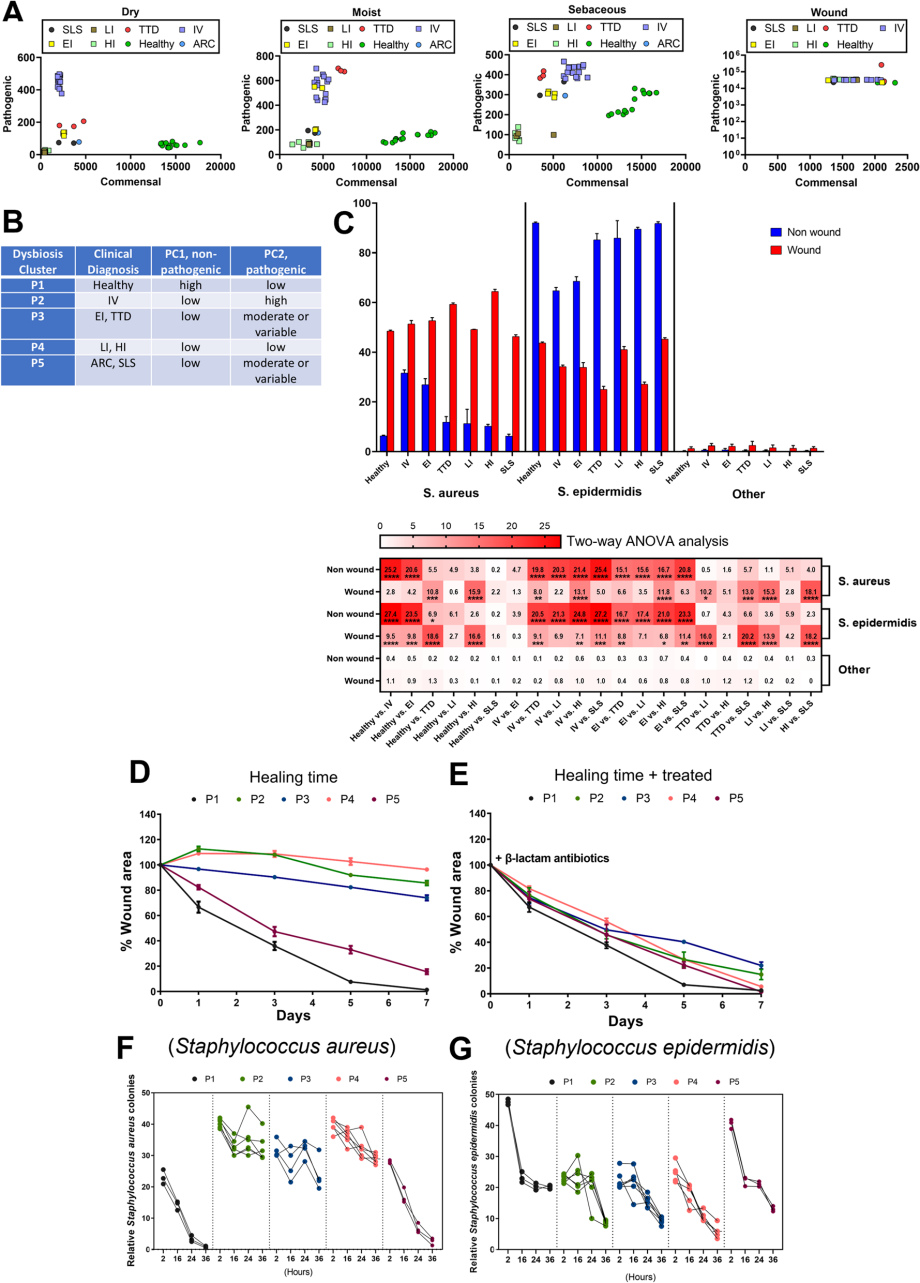
Dysbiosis clustering of CI patients and the pathogenic microbiome in wounded skin of CI patients. **A** PCoA plots showing beta diversity metrics in dry, moist, sebaceous, and wounded CI skin, colored according to CI type. **B** CI microbiome dysbiosis clusters and distribution of P1-P5 in each skin region, including Dry, Sebaceous (Seb), Moist, and Wounded were determined by principal co-ordinate analysis in panel A (PC1/commensal, PC2/pathogenic). Specifically, the sample composition for the Dry, Seb, and Moist areas encompassed 7 Healthy, 3 TTD, 1 ARC, 2 SLS, 8 HI, 3 LI, 15 IV, and 4 EI samples. Meanwhile, the sample composition for the Wound area consisted of 1 Healthy, 2 TTD, 1 SLS, 6 HI, 3 LI, 9 IV, and 2 EI samples. **C** (top) Dynamic changes in abundance of *S. aureus and S. epidermis* communities in non-wounded (blue) and wounded (red) skin from representative CI patients (IV, EI, TTD, LI, HI, SLS) compared with healthy controls. (bottom) Heatmap diagram showing each CI group compared to healthy controls, and each group compared to each other, using two-way ANOVA analysis. Results of each bar are expressed as mean ± SEM standard error. *p ≤ 0.05, **p ≤ 0.01, ***p ≤ 0.001, ****p ≤ 0.0001. **D**, **E** After wounding CI patients and healthy controls with 5 mm punch biopsy, healing time, measured as the change in percent wound area over 7 days, was delayed for the 4 CI dysbiosis clusters P2-P5 compared to healthy P1 controls in the absence of antibiotics (**D**), but in the presence of β-lactam antibiotics healing time improved to levels comparable to healthy controls in (**E**). **F**, **G** Measurement of relative colonies of *Staphylococcus aureus* (**F**) or *Staphylococcus epidermidis* (**G**) across the 5 dysbiosis clusters in wounded skin over 36 h

To investigate differences in pathogenic and commensal bacteria in healthy and CI patients across skin locations, the superficial swab OTU sequence data was subjected to principal co-ordinate analysis (PCoA). We identified dysbiosis clusters by collecting high-ratio non-pathogenic (principal component 1, PC1, x-axis) and high ratio pathogenic (principal component 2, PC2, y-axis) microbiomes observed in CI patients (Fig. 4A). Patients segregated into 5 distinct clusters following analysis of dry, moist, and sebaceous skin samples: cluster P1–healthy control subjects were high in PC1 and low in PC2; cluster P2–IV patients were high in PC2 and low in PC1 (*FLG* variants); cluster P3–EI and TTD patients were low in PC1 and variably elevated in PC2 in moist and sebaceous skin (*KRT1* and *ERCC2* variants, respectively); cluster P4–LI and HI patients were consistently lowest in both PC1 and PC2 across all skin sites (*TGM1* and *ABCA12* variants, respectively); and cluster P5–ARC and SLS patients were low in PC1 and variably elevated in PC2 in sebaceous skin only (*VPS33B* and *ALDH3A2* variants, respectively) (Fig. 4B). Principal component analysis (PCA) validated the PCoA (Additional file 6: Fig. S5).

### Wound infection impairs healing of CI skin

To investigate how the microbiota changes in CI patients when their skin is wounded, we introduced 5 mm punch biopsy wounds in a CI wound-cohort (n = 32; the ARC patient from P5 did not participate) and matched healthy controls (n = 5) and evaluated them for microbial infection, wound healing time, and inflammation. Using PCoA on collected OTU sequences, we demonstrated that wounds in CI skin are characterized by a major shift in the dysbiosis clusters of CI patients to a very high PC2 and low PC1 state (Fig. 4A, far right panel). This same shift occurred for the healthy controls.

Next, we delineated the relative abundance of microbial species in CI wounds. In non-wounded skin (close to wound region), *S. epidermidis* abundance was high for healthy and CI skin, with IV and EI patients having the least abundant *S. epidermidis* (Fig. 4C, blue, center panel). In non-wounded skin, *S. aureus* abundance was much lower than *S. epidermidis*, though higher among CI types than healthy controls (Fig. 4C, blue, left panel). In contrast, wounded skin demonstrated a large shift towards more abundant *S. aureus* (mean = 48.5% total relative abundance) for healthy and CI patients, with concomitant reduction in *S*. *epidermidis* (mean = 43.76% total relative abundance) (Fig. 4C, red), as supported by two-way ANOVA analysis (Fig. 4C bottom). *Streptococcus pyogenes* (mean = 3.3% total relative abundance) and low proportions of the zoonotic pathogen *Streptococcus suis* (0.3% relative abundance) were also observed more in wounded skin compared to healthy control skin (Additional file 7: Fig. S6A).

Some Gram-negative, facultative anaerobes such as *Pseudomonas aeruginosa* and *Campylobacter* demonstrated different relative abundance among dysbiosis clusters. For example, *P. aeruginosa* (2.15% relative abundance) showed the greatest elevated abundance in P2 (IV) and P3 (EI/TTD), a smaller elevation in P5 (SLS), but was not detected in P4 (LI/HI), compared to healthy controls (Additional file 7: Fig. S6A). *Campylobacter* (0.73% relative abundance) similarly showed elevated abundance in P2, P3, and the LI patients in P4. *Candida albicans* (0.82% relative abundance) was similar in abundance across healthy controls and IV patients (P2), but showed increased abundance in EI patients (P3) and SLS patients (P5), and decreased abundance in P4 and among TTD patients (P3). Methicillin-resistant *S. aureus* (MRSA) was most abundant in HI patients (P4) (Additional file 7; Fig. S6A and Additional file 8: Fig. S7A), and also observed in IV (P2). Using real-time PCR, we observed elevated HPV virus (1.45% relative abundance) in TTD (P3) and LI (P4) patients (Additional file 8: Fig. S7B).

To further evaluate whether the abundance of pathogenic microbes in CI patients (P2, P3, P4, and P5) impairs wound healing time versus healthy control subjects (P1), we wounded, by 5 mm punch biopsy, CI and control patients and observed their wound healing times in the absence or presence of oral β-lactam antibiotics (carbapenems and cephalosporins) (Fig. 4D, E). In the absence of antibiotics, the time required for wound healing was significantly delayed in P2, P3, and P4 CI patients compared to healthy controls (P1), with a smaller degree of wound healing delay in P5. In contrast, administration of β-lactam antibiotics led to nearly complete wound healing in all 4 CI dysbiosis clusters (P2–P5), with little change in the trajectory of healing time by healthy controls, indicating that the altered CI microbiome impairs wound healing.

To further characterize the response of wounded skin in CI patients, we evaluated expression levels of the anti-microbial peptide β-Defensin 2 (HBD-2) in healthy subjects (P1) and CI patients (P2–P5) (Additional file 7: Fig. S6B). We observed elevations of HBD-2 mRNA in wounded skin of CI patients from dysbiosis clusters P2, P3, and P4 compared to wounded skin of healthy controls (P1). CI patients in cluster P5, however, had similar levels of HBD-2 mRNA in wounded skin as the healthy controls. *S. aureus* colony counts (CFU) were significantly reduced in the wounded skin of healthy subjects (P1) and P5 CI patients after approximately 36 h of healing time (Fig. 4F). *S. aureus* colony counts remained high in P2–P4 CI patients after 36 h. *S. epidermidis* colony counts among healthy subjects (P1) and CI patients (P2–P5) were similarly reduced after a wound healing time of 24–36 h (Fig. 4G), although healthy subjects started and ended with higher overall *S. epidermidis* colony counts. *P. aeruginosa* colony counts were highly increased at 2 h and reduced back at 16 h in P3 and P4 CI patient wounds, and to lesser extend in P5, from 2 to 16 h healing time (Additional file 7: Fig. S6C).

In summary, CI patients showed: (i) distinct microbiota signatures with high dysbiosis, (ii) pathogenic bacterial levels linked to IV (P2) and EI and TTD (P3) patients, and (iii) generally low levels of all microbes present in HI and LI patients (P4). Viral susceptibility, such as to HPV or HBV/HCV, is particularly linked with CI patients (Additional file 15: Table S2). Breached wounds are a key determinant of all infectious microbe levels, mainly in the ratio of *S. aureus* to *S. epidermis* (Fig. 4C and Additional file 14: Table S1) and may drive the sepsis seen in CI patients. Therapies for CI skin can correct the ratio of *S. aureus* to *S. epidermis*, as we observed for TTD patients receiving phototherapy and IV patients receiving topical ammonium bituminosulfonate (Ichthammol) 20% ointment (Additional file 9: Fig. S8).

### Imbalance of immune and cytokine inflammation in CI patients

**Fig. 5:**
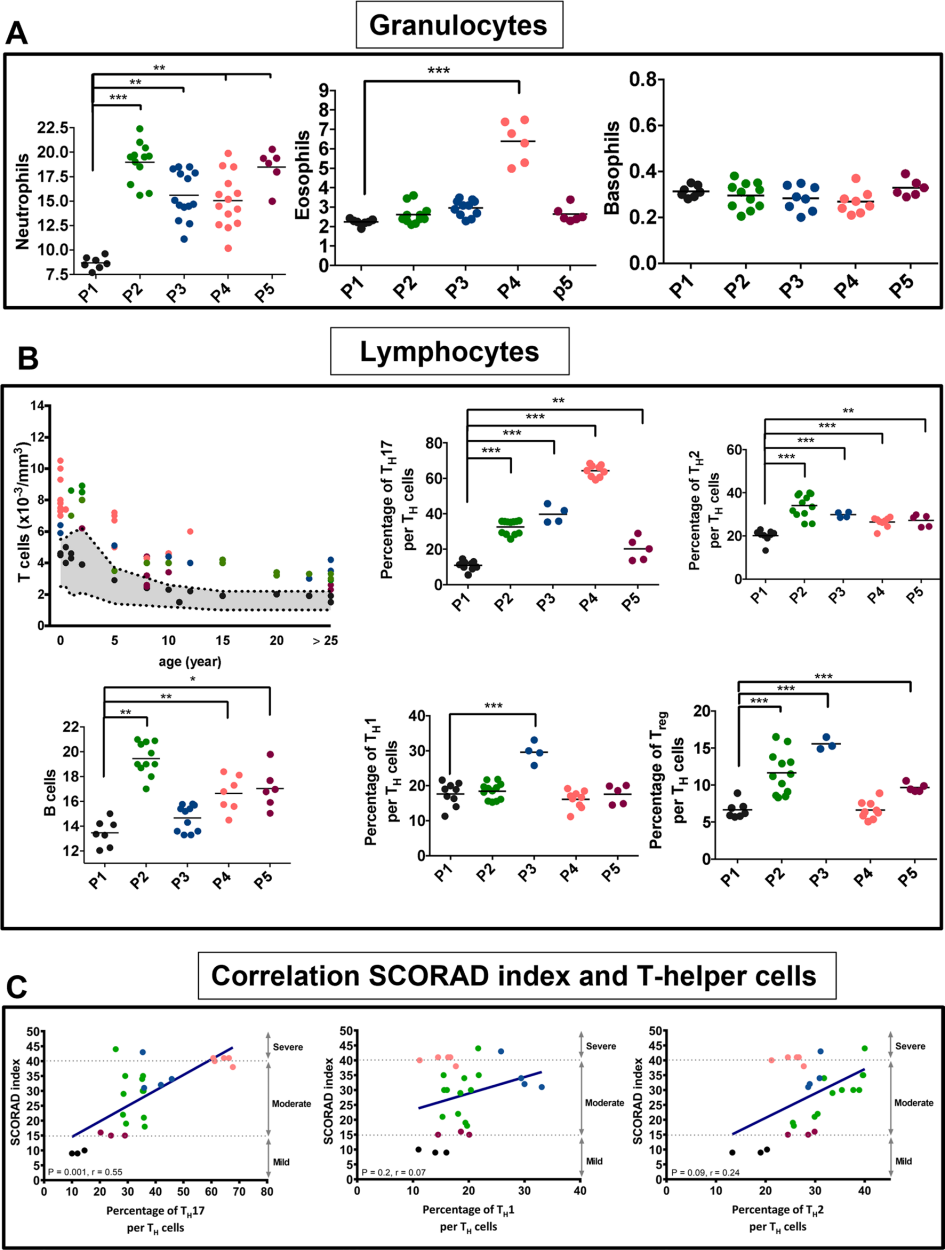
Immunotyping profile of each CI dysbiosis cluster. **A** Cell counts of granulocytes including neutrophils (left), eosinophils (center), and basophils (right) from healthy controls (P1) and CI patients (P2-P5) as determined by flow cytometry. Results are expressed in dot plot with each horizontal bar representing the mean of the group with statistical significance as *p ≤ 0.05, **p ≤ 0.01, ***p ≤ 0.001, ****p ≤ 0.0001. **B** Cell counts and relative percentages of lymphocytes including T, B, Th17, Th1, Th2, and Treg cells as determined by flow cytometry for healthy controls (P1) and CI patients (P2–P5). Results are expressed in dot plot with each horizontal bar representing the mean of the group with statistical significance as *p ≤ 0.05, **p ≤ 0.01, ***p ≤ 0.001, ****p ≤ 0.0001. **C** Correlation between SCORAD index with percentages of Th17, Th1, and Th2 cells in CI patients. Results are expressed in dot plot

To characterize inflammatory responses in CI patients across the dysbiosis clusters, we measured immune cell levels and cytokine responses. First, levels of granulocytes (neutrophils, eosinophils, and basophils) were measured from blood acquired from CI patients (no skin wounding; P2–P5) and healthy controls (P1). There was a statistically significant elevation in neutrophil count for all CI clusters (P2–P5) compared to healthy individuals (P1) (Fig. 5A, left panel). Interestingly, only CI patients from P4 (LI and HI) demonstrated a significant elevation in eosinophil counts (Fig. 5A, center panel), whereas there were no differences in basophils counts among CI patients compared to healthy controls (Fig. 5A, right panel).

Similarly, levels of T- and B- cells were quantified from blood from CI and healthy patients (Fig. 5B). Percentage of Th2^+^/CD4^+^ and Th17^+^/CD4^+^ cells are significantly increased in CI patients (P2, P3, P4, and P5) compared to healthy controls (Fig. 5B, upper right panels). Total Th1^+^/CD4^+^ cells are significantly increased in P3 CI patients, while Treg^+^/CD4^+^ cells are significantly increased in P2, P3, and P5 CI patients compared to healthy controls (P1) (Fig. 5B, bottom right panels). B cells were significantly induced in P2, P4, and P5 CI patients compared to P1 (Fig. 5B, bottom left panel). Importantly, CI patients were clinically subdivided into severe- (score > 40), moderate- (score = 15–40), and mild- (< 15) SCORAD index [27], a clinical tool for scoring the severity of atopic dermatitis/eczema; here it is applied to CI. SCORAD index in our CI cohort positively correlated with the percentage of Th17 and Th2 cells (Fig. 5C).

To further investigate our cohort for relevant genotype-immunologic phenotype correlations, Fisher exact tests and odds ratio calculations were performed for 6 immune cell types against the 7 genetic CI subtypes (Additional file 19: Table S6). We primarily observed statistically significant association of *FLG* mutation with neutrophil, B cell, Th1, Th2, Th17, and Treg counts; *ABCA12* mutation was also associated with neutrophil elevation.

**Fig. 6:**
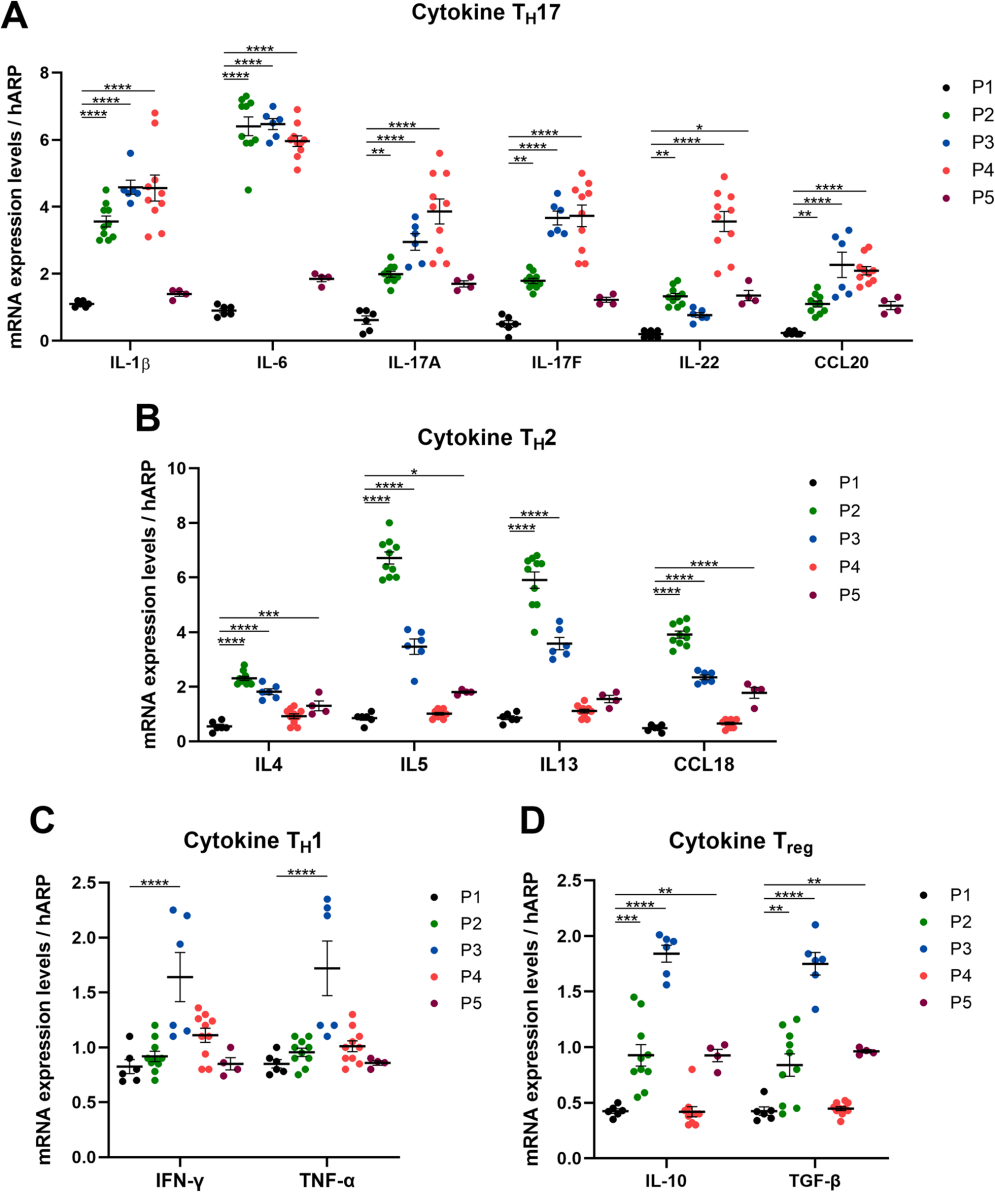
Immunocytokine profiles of CI patients analyzed across the CI dysbiosis clusters. **A**–**D** The measured mRNA expression levels of Th17 (**A**), Th2 (**B**), Th1 (**C**), and Treg (**D**) cytokines to housekeeping gene (hARP) across P1-P5 dysbiosis clusters. Results are expressed in bar graphs with overlapping dots representing each individual. Bars show mean ± SEM. Kruskal Wallis tests were used to compare the mRNA expression levels between groups with statistical significance as *p ≤ 0.05, **p ≤ 0.01, ***p ≤ 0.001, ****p ≤ 0.0001

Next, we measured circulating cytokine mRNA levels of Th1 (IFN-γ, TNF-α), Th2 (IL-4, IL-5, IL-13, CCL18), Th17 (IL-1β, IL-6, IL-17A, IL-17F, IL-22, CCL20) and Treg (IL-10, TGF-β) cytokines from peripheral-blood leukocytes (Fig. 6). CI patients (P2-P5) were generally found to have higher mRNA levels of Th17 cytokines compared with healthy control (P1) (Fig. 6A), except for notable insignificance in IL-22 in P3 and IL-1β, IL-6, IL-17A, IL-17F, CCL20 in P5 compared with healthy control (P1) in one-way ANOVA analysis (Additional file 10: Fig. S9A). Similarly, the mRNA level of IL-4, IL-5, IL-13 and CCL18 of Th2 was induced in CI patients (P2, P3 and P5) (Fig. 6B), but mRNA levels of IL-13 were not significantly elevated in P5 compared to P1 nor for any of the Th2 cytokines in P4 compared to P1 (Additional file 10: Fig. S9B). In addition, an increase in the mRNA level of Th1 cytokines IFN-γ and TNF-α was detected in P3 compared to P1 (Fig. 6C), and P3 levels of IFN-γ and TNF-α were significantly higher than the other dysbiosis clusters (P1, P2, P4, and P5) (Additional file 10: Fig. S9C). The mRNA levels of IL-10 and TGF-β Treg cytokines were increased in P2, P3, and P5 compared to P1, but not P4 (Fig. 6D). These cytokine elevations were significantly highest in P3 when compared with P1, P2, and P5 (Additional file 10: Fig. S9D).

**Fig. 7:**
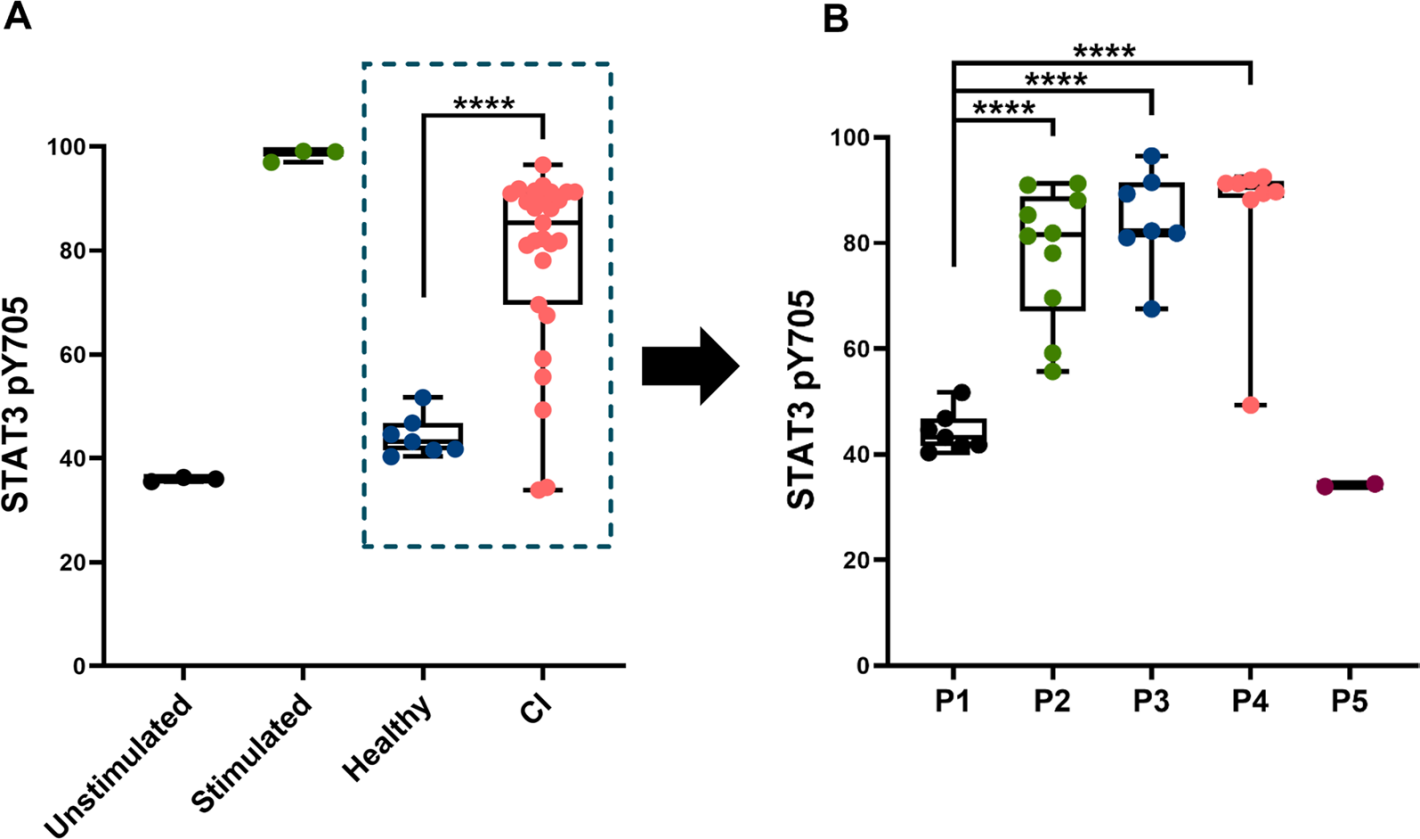
STAT3 pY705 levels as a marker for JAK/STAT signaling in CI patients. **A** Dot plot depicts STAT3 pY705 measured by flow cytometry in subcellular PBMCs. Line bar represents mean. Control samples were unstimulated or stimulated for 15 min with 400 μl Combo (cytokines (100 ng/ml), LPS (10 μg/ml; Sigma-Aldrich) or PMA (100 ng/ml; Sigma-Aldrich)). Healthy and CI patient samples were unstimulated by any substances. Results are plotted in dot plot showing median with interquartile range and statistical significance as *p ≤ 0.05, **p ≤ 0.01, ***p ≤ 0.001, ****p ≤ 0.0001. **B** Dot plot depicts STAT3 pY705 analyzed across the 4 CI dysbiosis clusters (P2, P3, P4, and P5) compared with healthy controls (P1). Results are plotted in dot plot showing median with interquartile range and SEM statistical significance as *p ≤ 0.05, **p ≤ 0.01, ***p ≤ 0.001, ****p ≤ 0.0001

To further analyze the signaling pathways in CI patients, we evaluated the levels of phosphorylated STAT3 Tyr705 (pY705) as a marker of Janus kinase (JAK) signaling activity in peripheral blood mononuclear cells (PBMCs) (Figs. 7 and Additional file 11: S10). Levels of STAT3 pY705 were significantly elevated in CI patients as a group compared to healthy controls (Fig. 7A). Analysis by dysbiosis cluster revealed profound elevations in STAT3 pY705 in P2, P3, and P4 compared with P1, but not in P5 (Fig. 7B).

Having established that CI patients generally display elevated neutrophils, Th17 cells, pro-inflammatory cytokines (IL-1β, TNF-α, IL-6, IL-10, and IL-17A/F), and activated JAK signaling/STAT3 p705 compared to healthy controls, we next investigated how the immune response is affected by wounding and wound healing in CI patients. Granulocyte and monocyte populations were analyzed by flow cytometry at baseline (0 h), 16 h, and 24 h after skin wounding using a 5 mm punch biopsy (Additional file 12: Fig. S11). Gr1^+^ granulocytes were elevated across P1-P5 at 16 h after wounding, but by 24 h post-wounding all returned closer to baseline except P2 and P3 (Additional file 12: Fig. S11B). This suggests prolonged inflammation in P2 and P3 CI patients post-wounding. Similarly, levels of Ly6^+^ monocytes increased across P1-P5 at 16 h post-wounding, and remained more elevated among P2, P3, and P5, and less so P4, compared to P1 at 24 h post-wounding (Additional file 12: Fig. S11C).

To further probe wound healing response in CI patients, we examined specifically the neutrophil, Th17/Treg, and dopamine-2 receptor (D2R) neural antibody counts in CI patients and healthy controls over 24 h, 48 h, and 7 days, respectively, post-wounding with a 5 mm punch biopsy (Additional file 12: Figs. S11D-F). There was a statistically significant higher proportion of neutrophils in CI patient clusters P2, P3, and P4 versus healthy controls (P1) at 24 h post-wounding, but not for CI cluster P5 (Additional file 12: Fig. S11D). The Th17/Treg count ratio was statistically elevated for all CI patients (P2-P5) compared to healthy controls at 48 h post-wounding (Additional file 12: Fig. S11E), indicating skin wounding in CI patients leads to a prolonged Th17 inflammatory response. Lastly, we examined D2R neural antibody as a measure of itch and observed statistically significant elevations in D2R levels before wounding as well as 7d post-wounding across CI patients in clusters P2-P5 (Additional file 12: Fig. S11F).

## Discussion

Patients with CI exhibit a range of symptoms, from mild, acute skin itching to chronic viral and bacterial infections of thick, scaly, fissured skin, with risk of severe or recurrent sepsis, and psychophysiological sequelae [28]. Prior to this study, there was limited data to understand the microbiome and immunological signatures accompanying the various ichthyoses, and even less data across races or ethnicities. We note after preparation of this report a microbiome study on 22 CI patients from 4 types (Congenital ichthyosiform erythroderma (CIE), LI, EI, Netherton’s Syndrome (NS)) was published [29]. Using WES we fully characterized the mutations underlying disease in 36 CI patients belonging to 7 CI types with Southeast Asian ethnicity, identifying 20 novel variants. Even with 36 patients and 7 subtypes, we acknowledge limitations in the overall participant size and CI subtypes in our analysis, primarily due to the rarity of CI genetic disorders, which makes validation of genotype–phenotype correlations difficult. Other limitations were access to care for patients in poor, rural Vietnam regions, and delays in data collection and analysis due to the COVID-19 pandemic. We demonstrated that the CI patient microbiota clusters into particular dysbiosis/patient groups according to ratios of pathogenic and non-pathogenic bacteria using 16S rRNA sequencing and PCoA/PCA. These microbiome signatures directly and differentially impacted wound healing and underlying immune cell populations and cytokine responses in flow cytometry and multiplex ELISA experiments. Our detailed microbiota characterization and immune phenotyping in a clinically and genetically well-defined patient cohort provides key disease insights that may improve CI clinical management.

Our microbiome analysis used 3 different skin sites (dry, moist, and sebaceous) because the healthy skin microbiome varies over body regions, 2 methods of quantification of microorganisms (cfu and OTU), and evaluated for bacteria, viruses, and fungi. Our study revealed marked differences in the prevalence of Actinobacteria, Firmicutes, Proteobacteria, and Bacteroidetes in the 3 different sites of skin. Similar to other skin disorders [30–32], our data showed low diversity of all phyla in dry, moist, and sebaceous skin sites of CI patients. Dermatologic conditions often share signatures of microbiome alterations [19]; for instance, *S. epidermidis* and *C. acnes* are less abundant than *S. aureus* within cutaneous inflammation in psoriasis patients [7]. Similarly, *S. aureus* is dominant in patients with ADAM17-deficiency related atopic dermatitis [33], while chronic *C. albicans* is often associated with impaired Th17 related genes [34].

PCA analysis of the microbiome data separated the CI patient cohort into 5 dysbiosis clusters (Fig. 4A, B), including the healthy subjects. Division of the 7 CI subtypes into 4 dysbiosis clusters generated insights into differences in the microbiological and immunological features between these diseases. In unwounded skin, most CI patients had lower abundance of *S. epidermidis*, normally a healthy skin commensal, as well as *C. acnes*. Reduced commensal *C. acnes* likely diminishes the skin barrier integrity of CI patients, since *C. acnes* produces short-chain fatty acids (propionic acid) that activate PPARα signaling leading to increased keratinocyte lipid synthesis, which ultimately supports ceramide production for the epidermal barrier [35]. In both moist and sebaceous skin, most CI patients had lower abundance of *S. epidermidis*, *S. hominis, S. capitis* (normally a healthy skin commensal) as well as *C. acnes* compared to the healthy group (P1), while the dry skin region of the CI patients showed no differences in abundance for these 4 microbes compared to the healthy group (P1). As reported before, the increase of *Bacteroides* species mostly contribute to soft tissue infection [36]. Interestingly, in our cohort study, *B. vulgatus* is significantly lower in moist and sebaceous skin of CI patients while *B. uniformis* is elevated on both dry and moist skin of IV and EI patients compared to the healthy group (P1).

The IV (P2) patients had all 3 skin regions significantly elevated in *S. aureus*, while only moist and sebaceous skin regions for *L. clevelandensis* and *Malassezia. spp* were elevated. Similar observations were made in areas close to a wound (non-wounded), where *S. aureus* was elevated higher in IV (P2) patients compared to the healthy group. Interestingly, the IV patients in our cohort who experienced sepsis had either frameshift or nonsense mutations in the N-terminal half of FLG whereas those having mutations toward C-terminal region of the FLG gene did not experience sepsis. The majority of patients with sepsis were infected with *S. aureus* (confirmed by blood culture and PCR) (Additional file 17: Table S4). This suggests IV patients may benefit from a combination of anti-staphylococcal and anti-fungal/anti-*Malassezia* therapies.

EI, HI, LI patients had elevation of *S. aureus* in dry skin compared to the healthy group (P1), but only the EI patients had the similar high elevation of *S. aureus* in the area close to a wound (non-wounded), while both HI and LI had similarly low level of *S. aureus* compared to the healthy group (P1). Interestingly, TDD patients had low level of *S. aureus* in all 3 skin regions and the area close to a wound (non-wounded), but the abundance of *S. aureus* in the wounded area is significantly higher. The EI (P3) patients had a significant increase in abundance of *Malassezia spp*. and *M. osloensis* on both moist and sebaceous regions compared to the healthy group (P1). This is different from a previous study [29], where EI patients had much lower abundance of *Malassezia spp*. Also different than previously reported [37], the increase of *Malassezia. spp* in the IV (P2), TTD (P3), and EI (P3) patients in our study did not limit the growth of the *S. aureus*. The sepsis cases in TTD and EI (P3) were most commonly caused by *S. aureus* (confirmed by blood culture and PCR) (Additional file 17: Table S4).

LI (P4) demonstrated notable decreases in abundance of *S. hominis*, *S. capitis*, *S. epidermidis*, *Malassezia spp*., *B. uniformis*, *B. vulgatus*, *C. acnes*, and *L. clevelandensis* across all 3 skin regions, and had non-statistical slight increase in *S. aureus* on dry skin. This finding contradicts prior observations [29], where the abundance of *S. capitis* species was increased for LI patients. However, our data show a similar increase in *S. aureus* on dry skin regions as reported before [29]. Despite non-significant elevation of *S. aureus* in non-wound area close to the wound or the dry skin region, P4’s sepsis cases were primarily caused by *S. aureus*, as well as *P. aeruginosa* and *S. pneumoniae* (confirmed by blood culture and PCR). *Moraxella* species were elevated in all CI clusters except P5 (ARC, SLS), whereas TTD was the only CI type to demonstrate elevated *B. pseudomallei*.

To better understand how the microbiome of CI patients reacts to skin wounding, we used a 5 mm punch biopsy as a model for skin wounding. While other models or approaches could be used for skin wounding, we found the punch method effective in elucidating differences in the microbiome and immune profile in wounded vs non-wounded CI skin. For example, while *S. aureus* abundance was slightly higher than healthy controls for all CI types in unwounded skin, it was substantially increased by skin wounding. There was a concomitant decrease in *S. epidermidis* in wounded CI skin. Importantly, there was a significant delay in wound healing time for CI clusters P2, P3, and P4, and a smaller delay for P5. We attribute this wound healing delay directly to the microbiome alteration because when given oral β-lactam antibiotics, the wound healing time for all CI clusters normalized closer to the healthy control. This data suggests that oral antibiotics, used with appropriate antibiotic stewardship in mind, may be an effective therapy for healing skin wounds in CI patients. Moreover, this data reinforces that injury or wounding in CI patients is a major risk factor for cutaneous *S. aureus* infection, which may correlate with risk of sepsis and neonatal lethality. Newborn CI patients in particular are at increased risk for health care–associated infections if the barrier of the skin is damaged. Similar to previous reports [38] where 75% of deaths had sepsis (n = 15 out of 20 deaths), 100% of HI patients who died in our study suffered sepsis (n = 4 out of 4), while 75% of total HI patients experienced sepsis (n = 6 out of 8); 4 HI patients had novel mutations in ABCA12, mostly truncating, resulting in the loss of the ABC2 domain of the ATP Binding Cassette Subfamily A Member 12. As noted above, IV patients in our cohort who experienced sepsis (Additional file 14: Table S1) had either frameshift or nonsense mutations in the N-terminal half of *FLG* whereas those having mutations more C-terminal did not experience sepsis. This indicates the type of mutation, its gene location, and its impact on protein production or structure can influence the CI disease course. IV is associated with earlier onset and worse severity of atopic dermatitis [39], which in turn is associated with higher *S. aureus* infections [33, 40].

Surprisingly, we found that despite high levels of *S. aureus* on the non-wounded areas close to wounded skin (Fig. 4C) and all 3 skin regions (Fig. 3H) of IV and EI patients, the amount of *S. aureus* observed in the wounded area of IV and EI patients was not higher than observed for other CI patients (Fig. 4C). In contrast, TTD and HI have relatively low abundance *S. aureus* on the non-wounded area close to the wound (Fig. 4C) and all 3 skin regions (Fig. 3H), but has the highest abundance for *S. aureus* in wounded skin (Fig. 4C). These findings may explain why the total rate of sepsis occurring among our IV and EI patients was 46% (n = 7 out of 15) and 25% (n = 1 out of 4), respectively, which were lower than the HI (75%, n = 6 out of 8), LI (66%, n = 2 out of 3), and TTD (100%, n = 3 out of 3) patient rates. Moreover, it is important to recognize that sepsis can be the major cause of death in certain CI types; our Vietnamese cohort had higher rates of sepsis among HI and TTD patients than reported previously for CI patients of non-Southeast Asian ethnicity: TTD (≤ 70%) [41] and HI (≥ 25%) [38]. This difference may be explained by the poor rural living conditions of our patient cohort or represent a physiologic difference among Asian skin. Lastly, how the skin becomes impaired may play a key role in how microbial infections progress in CI patients. The specific characteristics of the mutation driving the ichthyosis and its mechanism of altering the physical barrier may impact *S. aureus* behavior differently. For example, mutation of FLG in IV or KRT1 in EI may lower defense mechanisms against *S. aureus* but impair the epidermal barrier less than mutations in ABCA12 transport protein (HI), in ERCC2 (TTD) leading to faulty DNA repair mechanisms, and in TGM1 (LI) causing deficient stratum corneum cross-linking. Prior work showed *S. aureus* and *S. epidermidis* dysbiosis can promote skin inflammation in Netherton’s syndrome patients by altering skin protease function [42]. Further investigation is needed into how specific protein mutations affecting epidermal integrity impact the invasiveness of *S. aureus* and risk of sepsis, despite the low abundance of *S. aureus* in the skin microbiome composition.

**Fig. 8:**
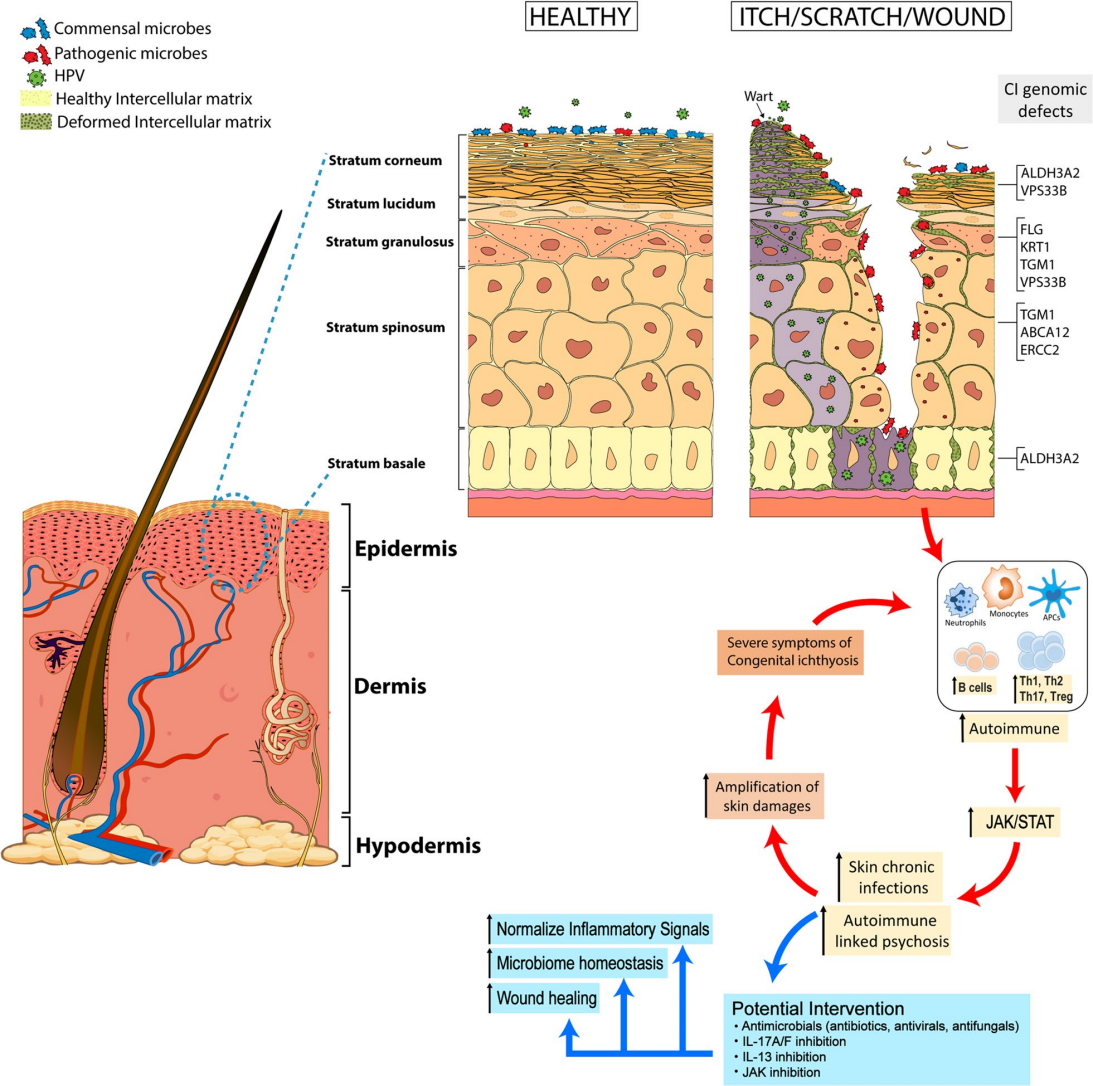
Model of congenital ichthyosis related to the barrier change in epidermis layers. The stratum corneum (SC) is the outermost layer of the epidermal barrier. The corneocytes of the SC act as bricks that form a hydrophilic wall that is surrounded by a lipophilic ‘‘mortar’’ made up of lipid lamellae, which fill the extracellular space. Genomics defects underlying CI diseases alter the epidermal barrier at various levels (SC, stratum spinosum (SS), and stratum basale (SB)), enabling pathogenic microorganisms to proliferate, invade, and cause microbial dysbiosis in the skin. In CI patients, as shown in this study, microbial dysbiosis and/or skin wounding amplifies Th17-driven inflammation in the skin, which can be associated with elevated JAK/STAT signaling

Immune profiling of the CI cohort with unwounded skin revealed several commonalities: all had elevations of neutrophils, normal basophil levels, and elevations of Th17 and Th2 T-lymphocytes. Differences, however, emerged among the CI clusters. For example, LI and HI patients (P4) were the only CI cluster with elevations in eosinophils. Th17 elevations were not uniform either: P4 had the highest percentage of Th17 cells, followed by P3 > P2 > P5. EI and TTD (P3) patients were the only CI cluster to have elevated percentages of Th1 cells, whereas P2, P3, and P5 had elevated Treg cells. At the cytokine level, it follows from the Th17 elevations that IL-1β, IL-6, IL-17A and IL-17F were also elevated based on mRNA expression levels, more so for P2, P3 and P4 than P5. For P2 and P3, IL-5 and IL-13 were elevated more than IL-4. Interestingly, upon wounding of the CI skin, there is a notable increase in the ratio of Th17 cells: Treg cells for at least 48 h, indicating a prolonged Th17 inflammatory response. Such potent ongoing inflammatory responses may create a continual inflammatory feedback loop limiting the ability of such patients to effectively heal following wounding (Fig. 8). Recent work investigating epigenetic control of inflammatory memory showed that STAT3 importantly recruits FOS-JUN, which is essential for driving chromatin accessibility and maintaining inflammatory memory in stem cells [43]. Our JAK/STAT signaling analysis showed elevated pSTAT3 for P2, P3, and P4 CI patients, suggesting a potential link between the observed Th17 and Th2 inflammation and STAT3 activation in CI patients. Notably, P5 CI patients had negligible pSTAT3 and correspondingly the lowest Th17 and Th2 immune profile.

Our data corroborates the heightened Th17 inflammatory response seen previously in CI patients [19–21, 44], which has stimulated investigations of the IL-17A inhibitor secukinumab as therapy for CI. While there are sporadic case reports of secukinumab improving a patient with autosomal recessive congenital ichthyosis harboring an *ABCA12* variant [45], as well as patients with Netherton’s syndrome [46, 47], a double-blind randomized controlled trial evaluating secukinumab in 4 types of CI patients compared to placebo failed to reduce disease severity or reduce Th17-related biomarkers [48]. Consistent with one study from 2019 [20], our data shows all CI dysbiosis clusters with elevated IL-17A and IL-17F. These two cytokines have synergistic/cooperative effects on one another [49], and clinical trial data for bimekizumab, a biologic with dual IL-17A and IL-17F inhibition, has achieved high rates of Psoriasis Area and Severity Index (PASI)-100 clearance [50]. Thus, it is plausible that treatment of CI patients with dual IL-17A/F inhibition will prove more effective than IL-17A inhibition alone.

The recent emergence of JAK inhibitors in dermatology, particularly those Food and Drug Administration-approved for atopic dermatitis (ruxolitinib, upadacitinib, abrocitinib), makes our finding of elevated JAK-STAT signaling in CI patient clusters P2, P3, and P4 intriguing. This suggests that JAK inhibitors, or even TYK2 inhibitors, might be effective in alleviating skin inflammation and barrier defects in many CI patient types. In fact, elevated JAK-STAT signaling activating the nitric oxide synthase (NOS2) pathway in HI has been documented and alleviated using tofacitinib in a 3D model of HI [51]. Moreover, itch was observed in 97.2% of our CI cohort, and JAK inhibitors to date have proved successful in reducing itch [52]. Another common driver of itch, IL-13 [53], was elevated for IV, EI, and TTD patients (P2 and P3), suggesting that the atopic dermatitis biologics tralokinumab or lebrikizumab, both IL-13 inhibitors, might prove useful in reducing itch and inflammation in some CI patients [54], or alternatively, the IL-4/IL-13 inhibitor dupilumab.

## Conclusions

This study represents the most comprehensive analysis of Southeast Asian congenital ichthyosis patients to date. Clinically, we followed our CI cohort for multiple years and documented relevant phenotypic features of 7 CI subtypes, ranging from the most common (hyperkeratosis) to the least (loss of heat). Furthermore, we established connection between mutations in skin barrier proteins and elevated rates of infection/sepsis in our cohort. We further determined that Southeast Asian CI patients have impaired response to wound healing, which can be mitigated by correcting microbial dysbiosis using oral antibiotics. The immune response among our CI cohort favored Th17 over Th2 cytokine pathways, which was driven predominantly by JAK/STAT3 activation. Given the link between STAT3 and FOS-JUN regulation of inflammatory memory in stem cells, these findings warrant further research into the role of stem cell inflammatory memory in CI pathogenesis. Ultimately, the microbiome dysbiosis clustering described here suggests that optimized treatment regimens for CI types will have to account for their heterogeneity from a genetic, microbiome, and immunological viewpoint.

## Materials and methods

### Sample collection for germline sequencing and microbiome

CI patients were recruited to participate in a study approved by the Institutional Review Board of UMP Ho Chi Minh City from December 2013 based on the Newton Researcher Link British Council project in compliance with all ethical regulations. From December 2016 we continued to recruit samples from the Nafosted project, supervised by Dr Nga Nguyen at Dalat University. Our study on ichthyosis and the skin microbiome includes individuals with various types of congenital ichthyosis: the patients were first screened for profound clinical phenotype of ichthyosis with signs and symptoms listed in Table 1 and confirmed through screening for genetically pathogenic variants associated with ichthyosis from a list of known genes, delineated in Table 2 (ABCA12, TGM1, FLG, KRT1, ERCC2, VPS33B, ALDH3A2). Patients had to be willing to adhere to the study's protocols. Excluded were individuals who had recently used antibiotics or antifungal medications, those with autoimmune, autoinflammatory, or other primary immunodeficiency genetic profiles, those with serious health issues, those who had undergone recent skin treatments or surgeries, and pregnant or breastfeeding individuals.

Written informed consent was obtained from all adult patients and the parents/guardians of all participating children (under 16-year-old). Subjects provided detailed medical history and underwent a physical examination at Children’s Hospital 2, Hung Vuong Maternity Hospital, UMP Medical Centre 3 and City Children’s Hospital, Vietnam. Follow-up schedule was separated in two periods (from 9/2013 to 9/2016, for DNA exome sequencing, and from 9/2018 to 12/2019, for wound healing test).

All samples were separated into 3 blood tubes (BD Biosciences); one EDTA vacutainer for germline DNA isolation, one CPT heparinized vacutainer for immune cell isolation, and one silicon-coated serum tube for cytokine analysis. All sterile Dacron swabs (Fisher Scientific, Sweden) from dry, moist, sebaceous, and lesioned skin were collected and DNA immediately extracted using the MO-BIO PowerSoil DNA isolation kit (MO-BIO Laboratories), otherwise, raw specimens were stored in a freezer (− 80 °C) until isolation of gDNA. Negative control swabs were also collected for each patient sample. Swabs were processed and cultured at the hospital department of microbiology following standard protocol to confirm the common skin bacteria as well as methicillin-resistant *S. aureus* infection processed [55]. All extracted DNA was quantified by NanoDrop and passed quality control testing prior to both WES or 16S-based metagenomic sequencing.

### Clinical whole exome sequencing

Clinical WES was described previously [56]. In brief, genomic DNA was extracted from peripheral blood using QIAamp DNA Blood Mini Kit (#51104, QIAGEN). DNA was subsequently examined for quality using DropSense96, Qubit 2.0, and TapeStation. The ideal range for these parameters were: chemical contaminants A260/A230: 1.50–2.50; protein contaminants A260/A280: 1.60–2.20; concentration > 1 ng/µL; DNA smear (50% > 1000 bp). Whole exome library preparation and sequencing was performed by Macrogen (South Korea), using Agilent SureSelect Human All Exon V5 (Agilent Technologies) on a NovaSeq 6000 Sequencing System (Illumina). Using paired FASTQ reads, FASTQC was used to obtain diagnostics such as Phred-score distribution along the reads, GC content distribution, read-length distribution, and sequence duplication level. After that, Trimmomatic was used for removing contaminated sequencing adapters; removing leading and trailing low quality or N bases below quality 3; scanning the read with a 4-base wide sliding window, cutting when the average quality per base drops below 15; and dropping reads below a length of 36. Pre-processed read pairs were then mapped to hg19 reference genomes (from UCSC) by BWA-mem. Additional processing included: MarkDuplicates by PICARD, BaseQualityScoreRecalibration by GATK. VCF files were generated with GATK HaplotypeCaller; filtrated by GATK VariantFiltration, SNP (QD < 2.0, FS > 60.0, MQ < 40.0, MQRankSum < − 12.5, ReadPosRankSum < − 8.0) and INDEL (QD < 2.0, FS > 200.0, ReadPosRankSum < − 20.0), respectively. Finally, ANNOVAR was used to intersect variant annotations from UCSC RefSeq, dbSNP 150, gnomAD, ESP6500, ExAC, 1000G, dbNSFPv3.5. The variant determination and classification are based on VarSome’s bioinformatics [57] that match the guideline of ACMG 2015 and all variants confirmed by Sanger sequencing.

### Microbiological investigations

DNA was isolated from frozen archived swab specimens with automatic platform Cobas4600 (Roche); then were loaded to array with AccuFill system. Specific strain was confirmed by blood cultures, bacterial cultures, and by multiplex PCR from TaqMan™ Comprehensive Microbiota Control (Thermo Fisher Scientific, Korea), and Antibiotic resistance genes (included sulfonamide resistance genes (sul1 and sul2), trimethoprim resistance genes (dfrA1, dfrA5), beta-lactam resistance genes (ampC, blaTEM, blaSHV and blaPSE-1) and tetracycline resistance genes (tet(B), and tet(M)) by TaqMan Assays (Thermo Fisher Scientific, Korea). Broad targeted genotyping Takara PCR-Realtime detection assay were customed by *Cutibacterium acnes*; *Lawsonella clevelandensis*; *Bacteroides uniformis*; *Bacteroides vulgatus*; *Staphylococcus capitis*; *Staphylococcus aureus*; *Staphylococcus hominis*; *Staphycococcus epidermidis*; *Morazella osloensis*; *Burkholderia pseudomallei*; *Malassezia* spp. Probes (SmartChip® Probe qPCR Master Mix) and TB Green® Advantage® qPCR Premix (Takara, Japan).

### Clinical confirmation of sepsis

The patients with sepsis were defined by having a high white blood cell count (> 11,000mm^3^), an elevated percentage of neutrophils (≥ 71%) and decreased percentage of lymphocytes (≤ 26%), and decrease platelet count (≤ 160,000mm^3^). Blood cultures (after 5 days culture) that were positive for *Staphylococcus aureus*, were considered as indication of bacterial infection. Additional criteria were an elevated lactate level (> 2 mmol/L), which can indicate tissue hypoxia and a more severe infection; and elevated Procalcitonin (PCT) level (> 0.5 ng/mL), which may indicate bacterial infection rather than a viral infection.

### Immunological investigations

One mL of whole blood was drawn into a TruCulture^®^ (Myriad RBM) tube containing TruCulture® media. Samples were incubated upright in a dry heat block at 37 °C for 30 h. Supernatant and cells were isolated and frozen for cytokines. T and B cell subsets were counted by 6-color TBNK BD TruCount (BD Biosciences) from patients and three or four age-matched healthy controls. mRNA cytokine levels were measured in cell-culture supernatants 2 h after in vitro stimulations by qPCR (Thermo Fisher).

### 16S rRNA sequencing and analysis

DNA isolation and library preparation to generate shot-gun metagenomic sequence data from microbiome followed a Macrogen protocol. The V1-V3 region of the 16S rRNA gene was amplified with previously described primer sets [1, 58]. ITS1 amplicons used are as previously reported [59]. The amplicon sequence data produced 15 million to 50 million 2 × 125-bp reads on an Illumina HiSeq platform (Macrogen). Raw sequence data were proceeded by Mothur pipeline to remove primers and barcodes. Chimeric reads from PCR artifacts were identified and trimmed by VSEARCH in Mothur. Secondary clusters were recruited into primary clusters. Noise sequences in clusters of size “a” or below were removed. The reads were clustered using a greedy algorithm into OTU clusters (including OTUs, Chao1, Shannon and Simpson index) at a user-specified OTU cutoff as previously described ([59]. Taxonomic assignment and diversity statistics were analyzed and visualized by Quantitative Insights into Microbial Ecology (QIIME). The qualified data then were classified to genus level using the k-nearest neighbor classifier against an updated custom ITS1 database. Phylotype for both 16S and ITS1 amplicon was analyzed to Mothur SOP. Quality processed reads not matching hg19 human reference were mapped against a database of 2,349 bacterial, 389 fungal, 4695 viral, and 67 archaeal reference genomes using Bowtie 2 (version 2.3.2)–very-sensitive parameter [60]. Read hit counts were normalized by genome size. To reduce the effect of low abundance misclassifications, we used a genome coverage cutoff of ≥ 1 for relative abundance and diversity calculations. Metagenomic reads not mapping to bacterial, fungal, or archaeal reference genomes were exported using the parameter of Bowtie 2 for de *novo* assembly.

Taxonomic classification of papillomaviruses is based on nucleotide similarity of the L1 gene51. The family *Papillomaviridae* contains 49 genera (with 5 genera representing human papillomaviruses), each of which is further divided into several species. To be designated as a new type, a single papillomavirus type cannot share > 90% similarity to any other known papillomavirus type in the L1 sequence. Papillomavirus types within a species share 71–89% nucleotide identity within the L1 gene and members of the same genus share > 60% L1 sequence identity.

### STAT3 signaling analysis

We collected PBM cells and extracted protein lysate for anti-STAT3 (Cell Signaling Technology, Cat#30835), anti-pSTAT3 (pY705, Cell Signaling Technology, Cat#9145), and anti-FAK1 analysis. We used the inhibitors FAK inhibitor 14 (Sigma-Aldrich) and Stattic (Sigma-Aldrich). Stattic was dissolved in dimethylsulfoxide (DMSO), while FAK inhibitor 14 was dissolved in water, at the desired concentrations and both stored at − 20 °C. High-throughput flow cytometry was used to detect cell subtypes containing STAT3 and STAT3 pY705 according to previous procedures [61, 62].

### R analysis

All statistical analyses were performed using R software. Data are represented as mean ± S.E.M. unless otherwise indicated. Spearman correlations of non-zero values were used for all correlation coefficients. Differences in OTU abundance between two groups were analyzed using White’s non-parametric *t*-test. For all boxplots, center lines represent the median while lower and upper box limits represent the first and third quartiles, respectively (interquartile range), whiskers represent the maximal values up to 1.5 times interquartile range, and all values beyond this range are defined as outliers. The non-parametric Kruskal–Wallis test was used to determine statistically significant differences between microbial populations. Unless otherwise indicated, P values were adjusted for multiple comparisons using the Bonferroni or FDR correction (by applying the adjust function in R using method = ‘bonferroni’ or ‘fdr’). Statistical significance was ascribed to an alpha level of the adjusted *P* ≤ 0.05. Similarity between samples was assessed using the Yue–Clayton theta similarity index with relative abundances of HPV species. The theta coefficient assesses the similarity between two samples based on (i) number of features in common between two samples, and (ii) their relative abundances, with θ = 0 indicating totally dissimilar communities and θ = 1 identical communities. Principal components were computed using the PCA function within the 'FactoMineR' package [63]. The visualization of all analysis results was carried out using the 'ggplot2' package [64].

### Genotype–phenotype association analysis

Fisher exact tests were utilized to establish the statistical significance of genotype–phenotype associations. Odds ratios (ORs) were computed to assess the association between genotype variables and the likelihood of phenotype events occurring; individuals included in the calculation must belong to the statistically satisfied sample pool number. The statistical analyses were performed using R version 4.2.3.

### Supplementary Information

**Additional file 1. **Supplemental Figure and Table Legends.

**Additional file 2. Figure S1. **Structural basis of TGM1 mutations in lamellar ichthyosis.

**Additional file 3. Figure S2. **Structural basis for keratin 1/keratin 10 mutations in epidermolytic ichthyosis.

**Additional file 4. Figure S3. **Structural basis for fatty aldehyde dehydrogenase mutations in Sjögren-Larsson syndrome.

**Additional file 5. Figure S4. **Structural basis for TTD mutations in trichothiodystrophy.

**Additional file 6. Figure S5. **PCA analysis for grouping.

**Additional file 7. Figure S6. **Microbiota and antimicrobial peptide changes in wounded CI skin compared to nonwounded skin.

**Additional file 8. Figure S7. **Presence of microbial and viral species in CI patients.

**Additional file 9. Figure S8. **Restoration of CI patient microbiome homeostasis with treatments of TTD and IV.

**Additional file 10. Figure S9. **One-way ANOVA analysis of cytokine expression levels across the 5 CI dysbiosis clusters.

**Additional file 11. Figure S10.** Procedure for determining JAK/STAT signaling profile in CI patients.

**Additional file 12. Figure S11.** Relative granulocyte and monocyte inflammation in CI patients following wounding of skin.

**Additional file 13. Figure S12.** Bacterium abundance plotted against CI gene mutation.

**Additional file 14**. Supplemental Table 1.

**Additional file 15**. Supplemental Table 2.

**Additional file 16**. Supplemental Table 3.

**Additional file 17**. Supplemental Table 4.

**Additional file 18**. Supplemental Table 5.

**Additional file 19**. Supplemental Table 6.

## Data Availability

The authors declare that all other data supporting the findings of this study are available within the paper and its supplementary information files.

## References

[1] Grice EA, Kong HH, Conlan S, Deming CB, Davis J, Young AC (2009). Topographical and temporal diversity of the human skin microbiome. Science.

[2] Lampe MA, Burlingame AL, Whitney J, Williams ML, Brown BE, Roitman E (1983). Human stratum corneum lipids: characterization and regional variations. J Lipid Res.

[3] Cogen AL, Nizet V, Gallo RL (2008). Skin microbiota: a source of disease or defence?. Br J Dermatol.

[4] Grice EA (2014). The skin microbiome: potential for novel diagnostic and therapeutic approaches to cutaneous disease. Semin Cutan Med Surg.

[5] Clausen ML, Agner T, Lilje B, Edslev SM, Johannesen TB, Andersen PS (2018). Association of disease severity with skin microbiome and filaggrin gene mutations in adult atopic dermatitis. JAMA Dermatol.

[6] Scharschmidt TC, List K, Grice EA, Szabo R, Program NCS, Renaud G (2009). Matriptase-deficient mice exhibit ichthyotic skin with a selective shift in skin microbiota. J Invest Dermatol.

[7] Byrd AL, Deming C, Cassidy SKB, Harrison OJ, Ng WI, Conlan S (2017). *Staphylococcus aureus* and *Staphylococcus epidermidis* strain diversity underlying pediatric atopic dermatitis. Sci Transl Med..

[8] Gonzalez ME, Schaffer JV, Orlow SJ, Gao Z, Li H, Alekseyenko AV (2016). Cutaneous microbiome effects of fluticasone propionate cream and adjunctive bleach baths in childhood atopic dermatitis. J Am Acad Dermatol.

[9] Paller AS, Kong HH, Seed P, Naik S, Scharschmidt TC, Gallo RL (2019). The microbiome in patients with atopic dermatitis. J Allergy Clin Immunol.

[10] Stevens ML, Gonzalez T, Schauberger E, Baatyrbek Kyzy A, Andersen H, Spagna D (2020). Simultaneous skin biome and keratinocyte genomic capture reveals microbiome differences by depth of sampling. J Allergy Clin Immunol.

[11] Dekio I, Sakamoto M, Hayashi H, Amagai M, Suematsu M, Benno Y (2007). Characterization of skin microbiota in patients with atopic dermatitis and in normal subjects using 16S rRNA gene-based comprehensive analysis. J Med Microbiol.

[12] Kong HH, Segre JA (2012). Skin microbiome: looking back to move forward. J Invest Dermatol.

[13] Paulino LC, Tseng CH, Strober BE, Blaser MJ (2006). Molecular analysis of fungal microbiota in samples from healthy human skin and psoriatic lesions. J Clin Microbiol.

[14] Fahlen A, Engstrand L, Baker BS, Powles A, Fry L (2012). Comparison of bacterial microbiota in skin biopsies from normal and psoriatic skin. Arch Dermatol Res.

[15] Statnikov A, Alekseyenko AV, Li Z, Henaff M, Perez-Perez GI, Blaser MJ (2013). Microbiomic signatures of psoriasis: feasibility and methodology comparison. Sci Rep.

[16] Clowry J, Irvine AD, McLoughlin RM (2019). Next-generation anti-*Staphylococcus aureus* vaccines: a potential new therapeutic option for atopic dermatitis?. J Allergy Clin Immunol.

[17] de Wit J, Totte JEE, van Mierlo MMF, van Veldhuizen J, van Doorn MBA, Schuren FHJ (2019). Endolysin treatment against *Staphylococcus aureus* in adults with atopic dermatitis: a randomized controlled trial. J Allergy Clin Immunol.

[18] Oji V, Tadini G, Akiyama M, Blanchet Bardon C, Bodemer C, Bourrat E (2010). Revised nomenclature and classification of inherited ichthyoses: results of the First Ichthyosis Consensus Conference in Soreze 2009. J Am Acad Dermatol.

[19] Paller AS, Renert-Yuval Y, Suprun M, Esaki H, Oliva M, Huynh TN (2017). An IL-17-dominant immune profile is shared across the major orphan forms of ichthyosis. J Allergy Clin Immunol.

[20] Malik K, He H, Huynh TN, Tran G, Mueller K, Doytcheva K (2019). Ichthyosis molecular fingerprinting shows profound T(H)17 skewing and a unique barrier genomic signature. J Allergy Clin Immunol.

[21] Czarnowicki T, He H, Leonard A, Malik K, Magidi S, Rangel S (2018). The major orphan forms of ichthyosis are characterized by systemic T-cell activation and Th-17/Tc-17/Th-22/Tc-22 polarization in blood. J Invest Dermatol.

[22] Dajnoki Z, Beke G, Mocsai G, Kapitany A, Gaspar K, Hajdu K (2016). Immune-mediated skin inflammation is similar in severe atopic dermatitis patients with or without Filaggrin Mutation. Acta Dermatol Venereol.

[23] Race E, Genetics WG (2005). The use of racial, ethnic, and ancestral categories in human genetics research. Am J Hum Genet.

[24] Rawlings AV (2006). Ethnic skin types: are there differences in skin structure and function?. Int J Cosmet Sci.

[25] Wan DC, Wong VW, Longaker MT, Yang GP, Wei F-C (2014). Moisturizing different racial skin types. J Clin Aesthet Dermatol.

[26] Brunner PM, Guttman-Yassky E (2019). Racial differences in atopic dermatitis. Ann Allergy Asthma Immunol.

[27] Schallreuter K, Levenig C, Berger J, Umbert J, Winkelmann R, Wegener L (1993). Severity scoring of atopic dermatitis: the SCORAD index. Dermatology.

[28] DiGiovanna JJ, Robinson-Bostom L (2003). Ichthyosis: etiology, diagnosis, and management. Am J Clin Dermatol.

[29] Tham K-C, Lefferdink R, Duan K, Lim SS, Wong XFCC, Ibler E (2022). Distinct skin microbiome community structures in congenital ichthyosis. Br J Dermatol.

[30] Byrd AL, Belkaid Y, Segre JA (2018). The human skin microbiome. Nat Rev Microbiol.

[31] Findley K, Grice EA (2014). The skin microbiome: a focus on pathogens and their association with skin disease. PLoS Pathog.

[32] Tirosh O, Conlan S, Deming C, Lee-Lin SQ, Huang X, Program NCS (2018). Expanded skin virome in DOCK8-deficient patients. Nat Med.

[33] Kobayashi T, Glatz M, Horiuchi K, Kawasaki H, Akiyama H, Kaplan DH (2015). Dysbiosis and *Staphylococcus aureus* colonization drives inflammation in atopic dermatitis. Immunity.

[34] Chang HW, Yan D, Singh R, Liu J, Lu X, Ucmak D (2018). Alteration of the cutaneous microbiome in psoriasis and potential role in Th17 polarization. Microbiome.

[35] Almoughrabie S, Cau L, Cavagnero K, O'Neill AM, Li F, Roso-Mares A (2023). Commensal *Cutibacterium acnes* induce epidermal lipid synthesis important for skin barrier function. Sci Adv.

[36] Wexler HM (2007). Bacteroides: the good, the bad, and the nitty-gritty. Clin Microbiol Rev.

[37] Li H, Goh BN, Teh WK, Jiang Z, Goh JPZ, Goh A (2018). Skin commensal *Malassezia globosa* secreted protease attenuates *Staphylococcus aureus* biofilm formation. J Invest Dermatol.

[38] Rajpopat S, Moss C, Mellerio J, Vahlquist A, Ganemo A, Hellstrom-Pigg M (2011). Harlequin ichthyosis: a review of clinical and molecular findings in 45 cases. Arch Dermatol.

[39] Bremmer SF, Hanifin JM, Simpson EL (2008). Clinical detection of ichthyosis vulgaris in an atopic dermatitis clinic: implications for allergic respiratory disease and prognosis. J Am Acad Dermatol.

[40] Kong H, Oh J, Deming C, Conlan S, Grice E, Beatson M (2012). Komarow HD; NISC Comparative Sequence Program, Murray PR, Turner ML Segre JA. Genome Res.

[41] Randall G, Kraemer KH, Pugh J, Tamura D, DiGiovanna JJ, Khan SG (2019). Mortality-associated immunological abnormalities in trichothiodystrophy: correlation of reduced levels of immunoglobulin and neutrophils with poor patient survival. Br J Haematol.

[42] Williams MR, Cau L, Wang Y, Kaul D, Sanford JA, Zaramela LS (2020). Interplay of staphylococcal and host proteases promotes skin barrier disruption in Netherton syndrome. Cell Rep.

[43] Larsen SB, Cowley CJ, Sajjath SM, Barrows D, Yang Y, Carroll TS (2021). Establishment, maintenance, and recall of inflammatory memory. Cell Stem Cell.

[44] Kim M, Mikhaylov D, Rangel SM, Pavel AB, He H, Renert-Yuval Y (2022). Transcriptomic analysis of the major orphan ichthyosis subtypes reveals shared immune and barrier signatures. J Invest Dermatol.

[45] Yogarajah J, Gouveia C, Iype J, Häfliger S, Schaller A, Nuoffer JM (2021). Efficacy and safety of secukinumab for the treatment of severe ABCA12 deficiency-related ichthyosis in a child. Skin Health Dis..

[46] Luchsinger I, Knöpfel N, Theiler M, Bonnet des Claustres M, Barbieux C, Schwieger-Briel A (2020). Secukinumab therapy for Netherton syndrome. JAMA Dermatol.

[47] Gan C, King E, Orchard D (2022). Secukinumab use in the treatment of Netherton's syndrome. Australas J Dermatol.

[48] Lefferdink R, Rangel SM, Chima M, Ibler E, Pavel AB, Kim H (2022). Secukinumab responses vary across the spectrum of congenital ichthyosis in adults. Arch Dermatol Res.

[49] Adams R, Maroof A, Baker T, Lawson ADG, Oliver R, Paveley R (2020). Bimekizumab, a novel humanized IgG1 antibody that neutralizes both IL-17A and IL-17F. Front Immunol.

[50] Freitas E, Blauvelt A, Torres T (2021). Bimekizumab for the treatment of psoriasis. Drugs.

[51] Enjalbert F, Dewan P, Caley MP, Jones EM, Morse MA, Kelsell DP (2020). 3D model of harlequin ichthyosis reveals inflammatory therapeutic targets. J Clin Investig.

[52] Lin DH, Nguyen C, Fleischer AB (2022). Time to meaningful clinical response in reduction of itch in atopic dermatitis. J Dermatol Treat.

[53] Du L-X, Zhu J-Y, Mi W-L (2022). Cytokines and chemokines modulation of itch. Neuroscience.

[54] Wollenberg A, Howell MD, Guttman-Yassky E, Silverberg JI, Kell C, Ranade K (2019). Treatment of atopic dermatitis with tralokinumab, an anti–IL-13 mAb. J Allergy Clin Immunol.

[55] Moran GJ, Krishnadasan A, Gorwitz RJ, Fosheim GE, McDougal LK, Carey RB (2006). Methicillin-resistant *S. aureus* infections among patients in the emergency department. N Engl J Med.

[56] Bui CB, Duong TTP, Tran VT, Pham TTT, Vu T, Chau GC (2020). A novel nonsense mutation of ERCC2 in a Vietnamese family with xeroderma pigmentosum syndrome group D. Hum Genome Var.

[57] Kopanos C, Tsiolkas V, Kouris A, Chapple CE, Albarca Aguilera M, Meyer R (2018). VarSome: the human genomic variant search engine. Bioinformatics.

[58] Methé BA, Nelson KE, Pop M, Creasy HH, Giglio MG, Huttenhower C, Gevers D, Petrosino JF, Abubucker S, Badger JH (2012). A framework for human microbiome research. Nature.

[59] Caporaso JG, Kuczynski J, Stombaugh J, Bittinger K, Bushman FD, Costello EK (2010). QIIME allows analysis of high-throughput community sequencing data. Nat Methods.

[60] Langdon WB (2015). Performance of genetic programming optimised Bowtie2 on genome comparison and analytic testing (GCAT) benchmarks. BioData Min.

[61] Fursov N, Gates IV, Panavas T, Giles-Komar J, Powers G (2011). Development and utilization of activated STAT3 detection assays for screening a library of secreted proteins. Assay Drug Dev Technol.

[62] Davies R, Vogelsang P, Jonsson R, Appel S (2016). An optimized multiplex flow cytometry protocol for the analysis of intracellular signaling in peripheral blood mononuclear cells. J Immunol Methods.

[63] Le S, Josse J, Husson F (2008). FactoMineR: an R package for multivariate analysis. J Stat Softw.

[64] Wickham H (2016). Data analysis. Use R.

