# Supplementary material for: Altered skin microbiome, inflammation, and JAK/STAT signaling in Southeast Asian ichthyosis patients
Source: Hum Genomics. 2024 Apr 16;18:38. doi: 10.1186/s40246-024-00603-x (PMC11022333; doi:10.1186/s40246-024-00603-x)
Supplement: Supplementary file 1 — Additional file 1. Supplemental Figure and Table Legends. [file 40246_2024_603_MOESM1_ESM.docx]

**Supplemental Figure** **and Table Legends**

**Figure S1. Structural basis of TGM1 mutations in lamellar ichthyosis.** A structural model of TGM1 was produced based on coagulation factor XIII (Protein Data Bank (PDB) Accession Code 1F13, which had a higher sequence identity and better GMQE score than the transglutaminase 2 structure). The TGM1 model overlays well with the TGM2 structure (PDB 3LY6) with the catalytic triad (C377, H436, D459, within red circle) well-conserved. R323 is highly conserved in the transglutaminase family and the R323W mutation increases the acidity of the protein surface in a pocket very close to the active site, which is itself very acidic (Herman et al., 2009). TGM1 is cleaved from a low activity to high activity form between S92 and R93, thus the R93Q mutation disrupts this process (Kim et al., 1995).

**Figure S2. Structural basis for keratin 1/keratin 10 mutations in epidermolytic ichthyosis.**  (A) A structural model of the K1/K10 helix 1A heterocomplex was produced using the K5/K14 helix 2B crystal structure (PDB Code 3TNU) as a template based on GMQE score. L187F mutation is at the N-terminus of the 1A domain, specifically located at the heterodimer interface. The mutation likely destabilizes the heterodimer due to increased, bulky size of the amino acid at the mutated position causing steric clashes with the K1 backbone as well as the side chains of L153^K1^, L157^K1^, and F191^K10^. (B) The published crystal structure of the K1/K10 helix 2B heterocomplex (PDB Code 4ZRY) was used to investigate the effects of missense mutations in this keratin region. The R432G mutation eliminates a surface-exposed basic residue, thereby reducing positive surface charge and increasing the acidity of the region and likely disrupting an interaction with another keratin molecule required for mature filament assembly. The G488V mutation occurs at the C-terminus in the highly conserved TYR*LLEGE motif known to be critical for intermolecular interactions (Wilson et al., 1992; Lomakin et al., 2020). The G488V mutation is thus likely to disrupt higher order filament assembly.

**Figure S3. Structural basis for fatty aldehyde dehydrogenase mutations in Sjögren-Larsson syndrome.**

*ALDH3A2* encodes the membrane-bound fatty aldehyde dehydrogenase (FALDH), which converts long-chain fatty aldehydes to fatty acids. The published x-ray crystal structure of FALDH (PDB 4QGK) was used to investigate missense mutations in *ALDH3A2*. The P315S mutation is predicted to destabilize the folding of the protein as it removes a proline in a loop, changing the orientation of the loop. Furthermore, this mutation introduces a polar serine into an otherwise hydrophobic region buried in the protein, resulting in entropic destabilization of this region. The L456T mutation, which was observed in the same patient with the P315S mutation, also causes disruption of hydrophobic interactions. These two mutations occurring concomitantly destabilize the protein structure to reduce its catalytic activity. The L456T mutation is also significant because it occurs in a helix thought to act as a gatekeeper for substrates. Another individual disease-causing mutation, K431Q, reduces the positive charge at the surface of the enzyme. The charged residues in this region are conserved in the FALDH protein family and have the function of binding the negatively charged head groups of the lipid bilayer. Consequently, the charge reduction caused by the K431Q mutation may reduce substrate affinity and thus impede enzyme activity (Keller et al., 2014).

**Figure S4. Structural basis for TTD mutations in trichothiodystrophy**. *ERCC2* encodes the excision repair protein TTD, which makes up one subunit in the general transcription and DNA repair factor IIH (TFIIH) complex. TTD has helicase and ATP hydrolysis activity, making it an important part of the TFIIH complex (Yan et al). The structure of DNA-bound TFIIH, including TTD, has been determined by cryo-EM (PDB 6RO4) and was used to model the disease-causing mutations Y197A and R683Q. The Y197A mutation may alter the folding of the protein as it significantly decreases residue size and eliminates some hydrophobic interactions (with P169, L105). This may have a noticeable impact on protein function because it occurs one residue away from R196, which directly interacts with the DNA substrate. Similarly, R683 interacts with the DNA backbone. Elimination of the positive arginine charge is expected to reduce the enzyme interaction with the negatively-charged DNA molecule.

**Figure S5. PCA analysis for grouping.**

We took the total microbiome OTU reads of individual patients and analyzed them using R FactoMineR package to determine the principal component analysis for the groups (P1, P2, P3, P4, P5). We used the R package ggplot2 to draw the PCA and perform clustering analysis of the groups for different skin regions: A. dry skin, B. moist skin, C. sebaceous skin, D. wounded skin.

**Figure S6. Microbiota and antimicrobial peptide changes in wounded CI skin compared to non-wounded skin.**

A. Dynamic changes in abundance of select microbial community species in non-wounded (left) and wounded (right) skin of representative CI patients (IV, EI, TD, LI, HI, SLS) compared with healthy controls.

B. Level of innate antibacterial β-defensin 2 (mRNA HBD-2) in wounded CI skin (P2, P3, P4, and P5) compared to wounded skin from healthy controls (P1). Results are expressed in dot plot with each horizontal bar representing the mean of the group.

C. Measurement of relative colonies of *Pseudomonas* aeruginosa across the 5 CI dysbiosis clusters (P1-P5) for 16 hours after skin wounding.

**Figure S7. Presence of microbial and viral species in CI patients.**

A. Bacterial culture of Trychophyton, *C. albicans* and MRSA of CI patients. Samples collected from IV2 infected by Trychophyton and *C. albicans* and from HI6 infected with MRSA, which caused septic shock.

B. Detection of human papillomavirus (HPV) subtypes 6, 16, and 12 for TTD2 patient.

**Figure S8. Restoration of CI patient microbiome homeostasis with treatments of TTD and IV.**

Microbiome changes during therapeutic treatment for TTD and IV subtypes of congenital ichthyosis were examined. Participants who received antibiotics or experienced side effects after treatment were excluded. Eight healthy control samples were collected from intact skin on the face, arm, and leg. Relative abundance of *S. aureus*, *S. epidermis*, *C. acnes*, and *B. pseudomallei* colonization was examined with microbiological real-time PCR of the 16S-rRNA test.

A. Three TTD patients received narrow-band UVB phototherapy treatment in a mean dose of 25.5 J/cm2 twice weekly for 6 weeks (8 focal lesions on face-nose, forehead, and cheek were collected). Specimens were analyzed from TTD patients prior to treatment, after 2 weeks phototherapy, and after 6 weeks phototherapy to evaluate the targeted species abundance. Phototherapy increased commensal organisms *S. epidermidis* and *C. acnes* closer to healthy levels, while decreasing *B. pseudomallei*. Relapse of skin lesions on TTD patients occurred 6 months after completing phototherapy. Results are expressed in dot plot with median and interquartile range with statistical significance as **p* ≤ 0.05, ***p* ≤ 0.01, ****p* ≤ 0.001, *****p* ≤ 0.0001.

B. Twelve IV patients (16 focal lesions on leg/arm and on face were collected) applied once daily ointment treatment (ammonium bituminosulfonate (Ichthammol) 20% with zinc oxide (Ichthopaste® bandage)) for 2 weeks along with oral supplemental 1000mcg Vitamin B12. Specimens were taken from IV patients prior to treatment and 2 weeks after treatment to evaluate the targeted species abundance. Topical ointment therapy after 2 weeks reduced *S. aureus* in IV patients and restored *S. epidermidis* and *C. acnes* abundance to levels close to healthy controls. Results are expressed in dot plot with median and interquartile range with statistical significance as **p* ≤ 0.05, ***p* ≤ 0.01, ****p* ≤ 0.001, *****p* ≤ 0.0001.

**Figure S9. One-way ANOVA analysis of cytokine expression levels across the 5 CI dysbiosis clusters.**

Heatmap diagrams showing relative expression of Th17 (A), Th2 (B), Th1 (C), and Treg (D) cytokine levels for each CI group (P2-P5) compared to healthy controls (P1), and each CI group compared each other. **p* ≤ 0.05, ***p* ≤ 0.01, ****p* ≤ 0.001, *****p* ≤ 0.0001

**Figure S10. Procedure for determining JAK/STAT signaling profile in CI patients.**

High-throughput subtyping of cells via flow cytometry enabled assessment following the protocol of Davies et al., 2016. Cryopreserved PBMCs were thawed and rested at 37 °C with serum free media, 5% CO2 for 11.5 h to reduce basal phosphorylation levels before plated into Megablock® 96 well plate in a final cell concentration of 1 × 106 cells/ml. Control samples were unstimulated or stimulated for 15 min with 400 μl Combo (cytokines (100 ng/ml), LPS (10 μg/ml; Sigma-Aldrich) or PMA (100 ng/ml; Sigma-Aldrich)). The patient samples were unstimulated by any substances. Next, samples were fixed, permeabilized, and barcoded with staining of pacific blue (PB) and pacific orange (PO) dyes. Single cells were gated based on their forward scatter area (FSC-A) and forward scatter width (FSC-W), followed by intact cells based on side scatter area (SSC-A) and FSC-A. The different stimulation conditions were then identified through the intensities of their PB and PO stains. Cell subtypes were identified based on their FSC-A and SSC-A scatter properties as either monocytes or lymphocytes. Lymphocytes were then subtyped based on surface antigens including B cells (CD19 +), T cells (CD3 +/ CD4 +, CD8 + CD56 −), NK cells (CD3 − CD56 +). Cells within each subtype were analysed based on the change of normalized median fluorescence intensity in each stimulation condition relative to the unstimulated reference samples. STAT3 and STAT3 pY705 levels in monocytes, CD3+ T-cells, B cells, and NK cells were analysed in scatter graphs and heat maps.

**Figure S11. Relative granulocyte and monocyte inflammation in CI patients following wounding of skin.**

A. Flow cytometry gating strategy for CD11b^hi^, GrR1^+^ granulocytes, and CD11b^hi^, Ly6C^+^ monocytes

B. Relative CD11b^hi^Gr1^+^ granulocyte populations followed longitudinally over 24 hours following wounding for all 5 CI dysbiosis clusters. Results expressed in dot plot with each horizontal bar representing the mean of the group.

C. Relative CD11b^hi^Ly6C^+^ monocyte populations followed longitudinally over 24 hours following wounding for all 5 CI dysbiosis clusters. Results expressed in dot plot with each horizontal bar representing the mean of the group.

D. Percentage of neutrophils counted over 24 hours compared in each CI dysbiosis population following wounding. Results are expressed in dot plot showing median and interquartile range with statistical significance as **p* ≤ 0.05, ***p* ≤ 0.01, ****p* ≤ 0.001, *****p* ≤ 0.0001.

E. Percentage of Th17/Treg cells counted over 48 hours compared in each CI dysbiosis population following wounding. Results are expressed in dot plot showing median and interquartile range with statistical significance as **p* ≤ 0.05, ***p* ≤ 0.01, ****p* ≤ 0.001, *****p* ≤ 0.0001.

F. Number of auto-antibody D2R counted over 7 days compared in each CI dysbiosis population following wounding. Results are expressed in dot plot showing median and interquartile range with statistical significance as **p* ≤ 0.05, ***p* ≤ 0.01, ****p* ≤ 0.001, *****p* ≤ 0.0001.

**Supplement Table 5 & 6:**

Insights into associations of phenotypes and genotypes. The data are presented, encompassing odds ratios (OR) along with their corresponding 95% confidence intervals (95% CI), and p-values obtained from Fisher's Exact Test to evaluate statistical associations, p<0.05 was considered statistically significant. Sample sizes (n) are explicitly indicated for each group, with "No." representing the number of present events out of the total number of samples in each group within the statistically satisfied sample pool used in the odds ratio analysis.

**Supplementary Citations**:

Davies R, Vogelsang P, Jonsson R, & Appel S. An optimized multiplex flow cytometry protocol for the analysis of intracellular signaling in peripheral blood mononuclear cells. Journal of Immunological Methods 2016;436:58-63.

Herman ML, Farasat S, Steinbach PJ, Wei MH, Toure O, Fleckman P, Blake P, Bale SJ, Toro JR. Transglutaminase-1 (TGM1) gene mutations in autosomal recessive congenital ichthyosis: summary of mutations (including 23 novel) and modeling of TGase-1. Hum Mutat 2009;30(4):537-547.

Keller MA, Zander U, Fuchs JE, Kreutz C, Watschinger K, Mueller T, Golderer G, Liedl KR, Ralser M, Krautler B, Werner ER, Marquez JA. A gatekeeper helix determines the substrate specificity of Sjogren-larsson syndrome enzyme fatty aldehyde dehydrogenase. Nat commun 2014;5:4439.

Kim SY, Chung SI, Steinert PM. Highly active soluble processed forms of the transglutaminase 1 enzyme in epidermal keratinocytes. J Biol Chem 1995;270:18026-18035.

Lomakin IB, Hinbest AJ, Ho M, Eldirany SA, Bunick CG. Crystal structure of keratin 1/10(C401A) 2B heterodimer demonstrates a proclivity for the C-terminus of helix 2B to form higher order molecular contacts. Yale J Biol Med 2020;93:3-17.

Wilson AK, Coulombe PA, Fuchs E. The roles of K5 and K14 head, tail, and R/K L L E G E domains in keratin filament assembly in vitro. J Cell Biol 1992;119:401-414.

Yan C, Dodd T, He Y, Trainer JA, Tsutakawa SE, Ivanov I. Transcription preinitiation complex structure and dynamics provide insight into genetic diseases. Nat struct mol biol 2019;26:397-406.
