# Supplementary figures and images for: Altered skin microbiome, inflammation, and JAK/STAT signaling in Southeast Asian ichthyosis patients
Source: Hum Genomics. 2024 Apr 16;18:38. doi: 10.1186/s40246-024-00603-x (PMC11022333; doi:10.1186/s40246-024-00603-x)

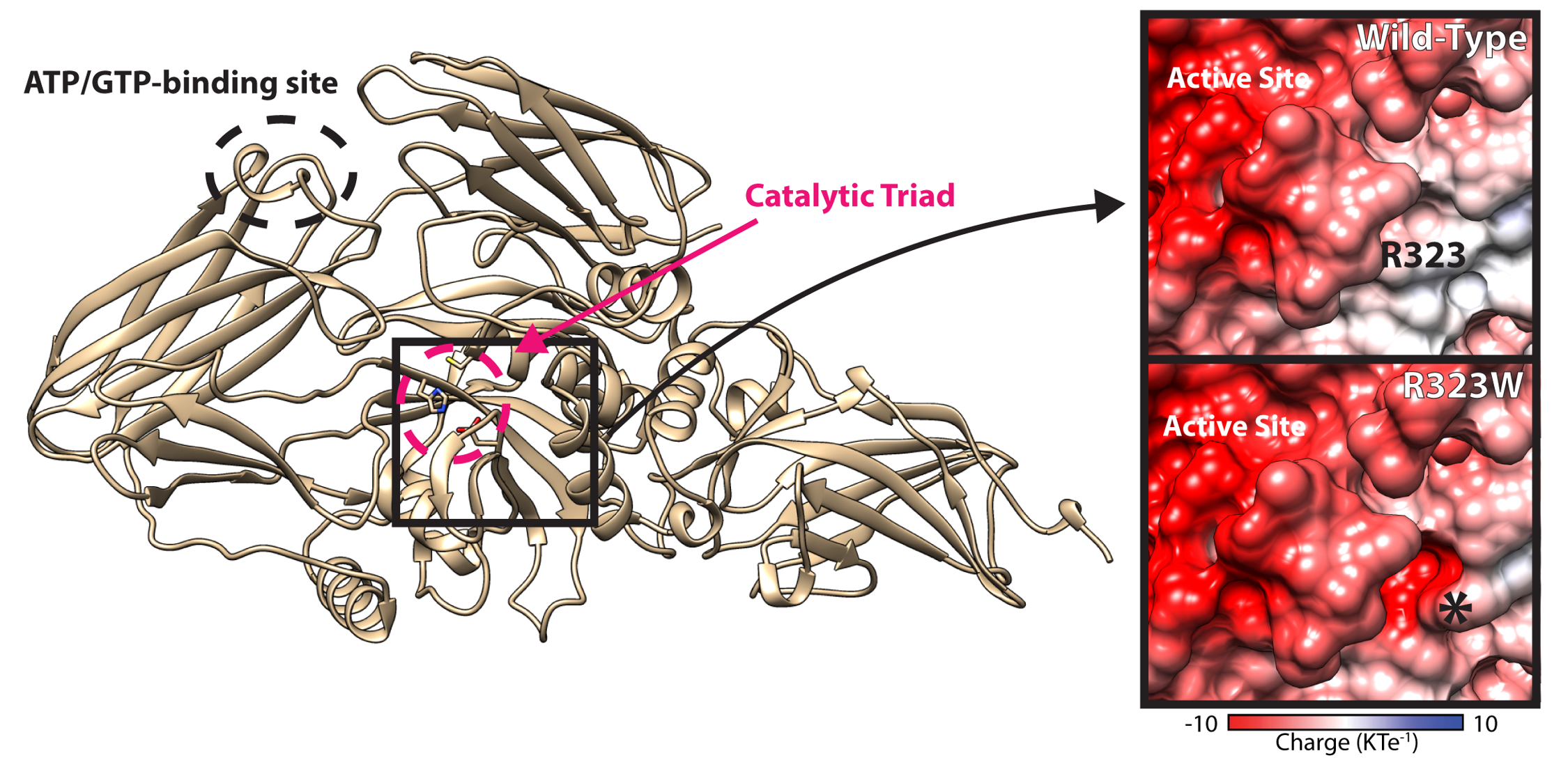

Supplement: Supplementary file 2 — Additional file 2. Figure S1. Structural basis of TGM1 mutations in lamellar ichthyosis. [file 40246_2024_603_MOESM2_ESM.tif]

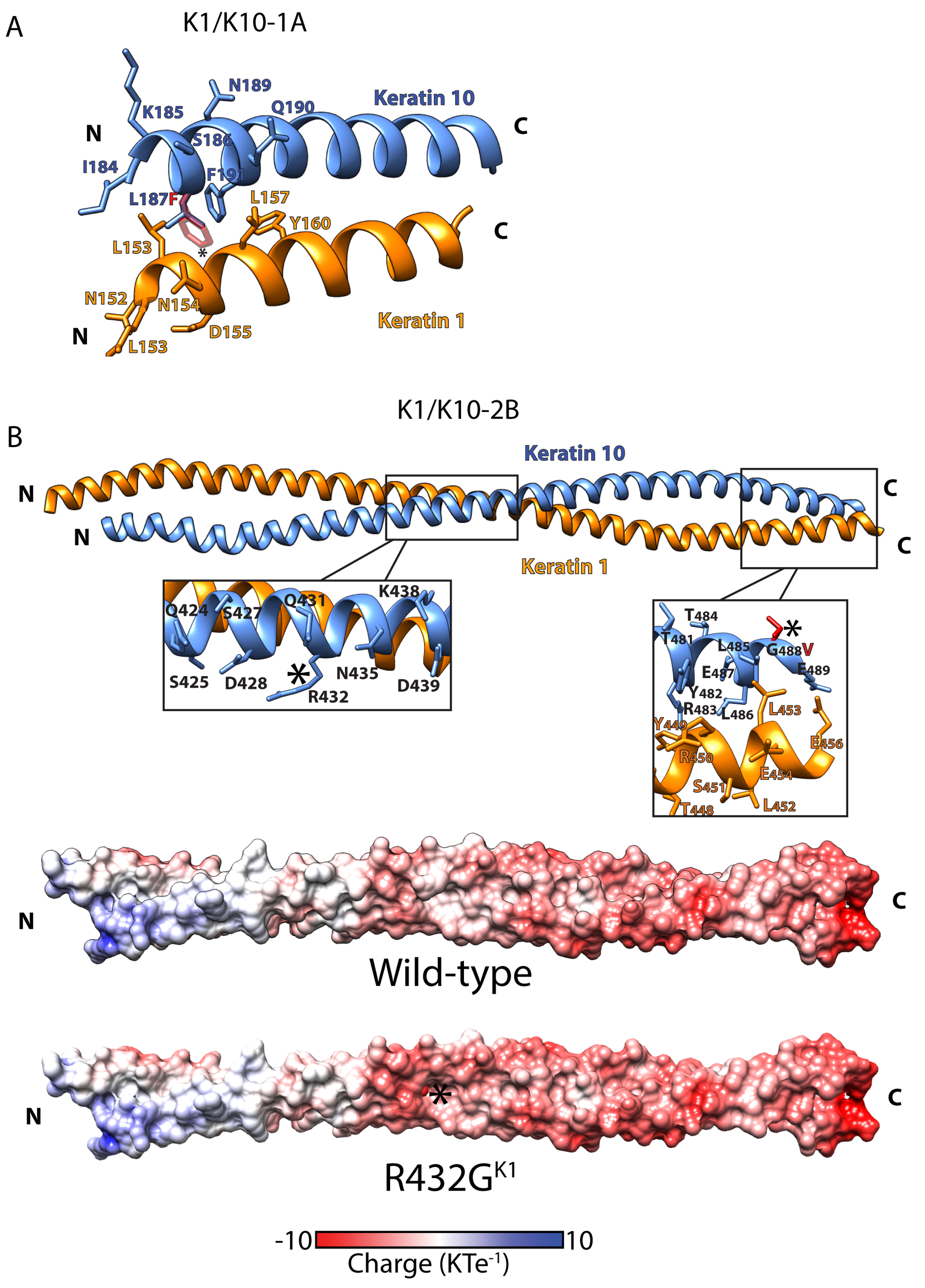

Supplement: Supplementary file 3 — Additional file 3. Figure S2. Structural basis for keratin 1/keratin 10 mutations in epidermolytic ichthyosis. [file 40246_2024_603_MOESM3_ESM.tif]

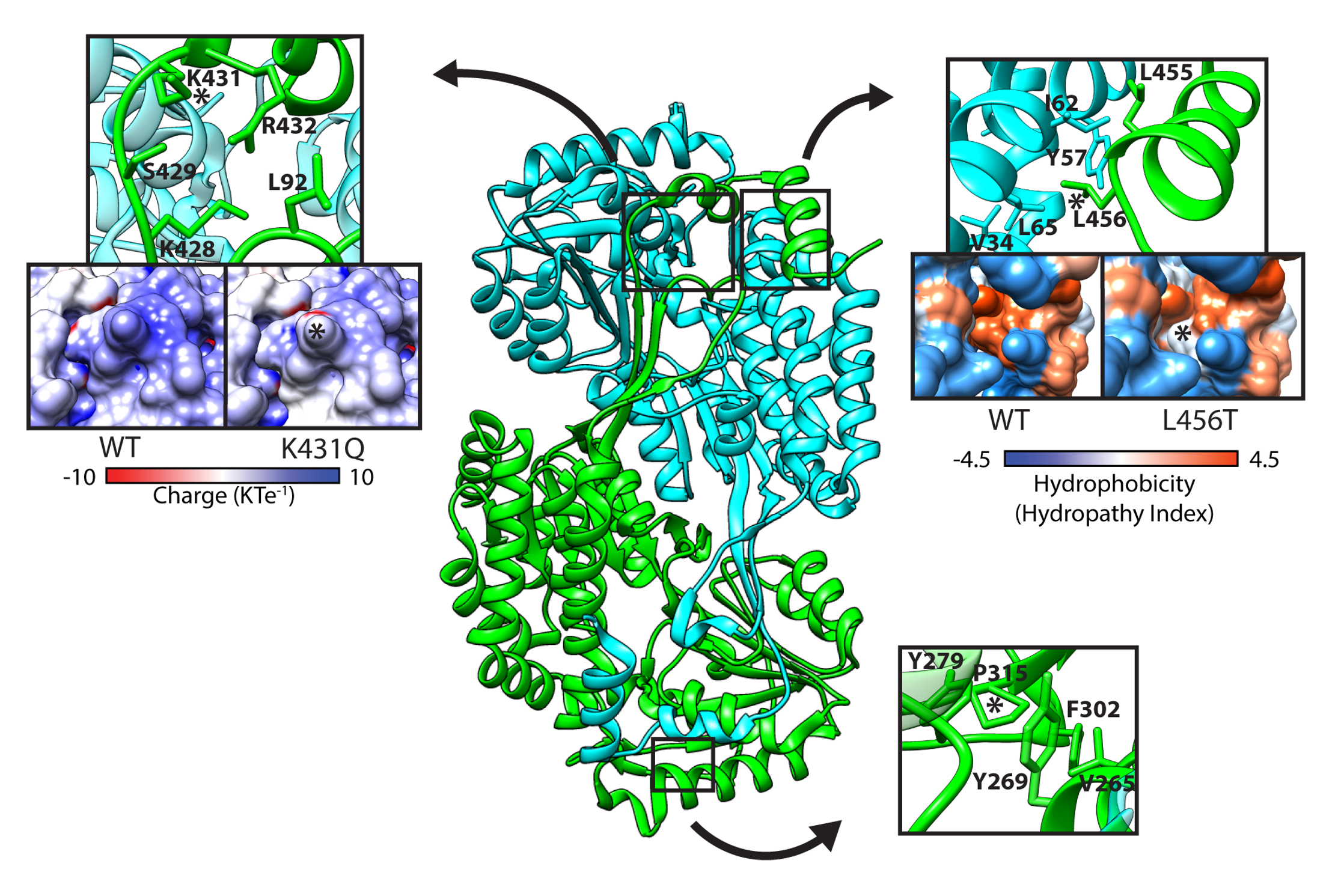

Supplement: Supplementary file 4 — Additional file 4. Figure S3. Structural basis for fatty aldehyde dehydrogenase mutations in Sjögren-Larsson syndrome. [file 40246_2024_603_MOESM4_ESM.tif]

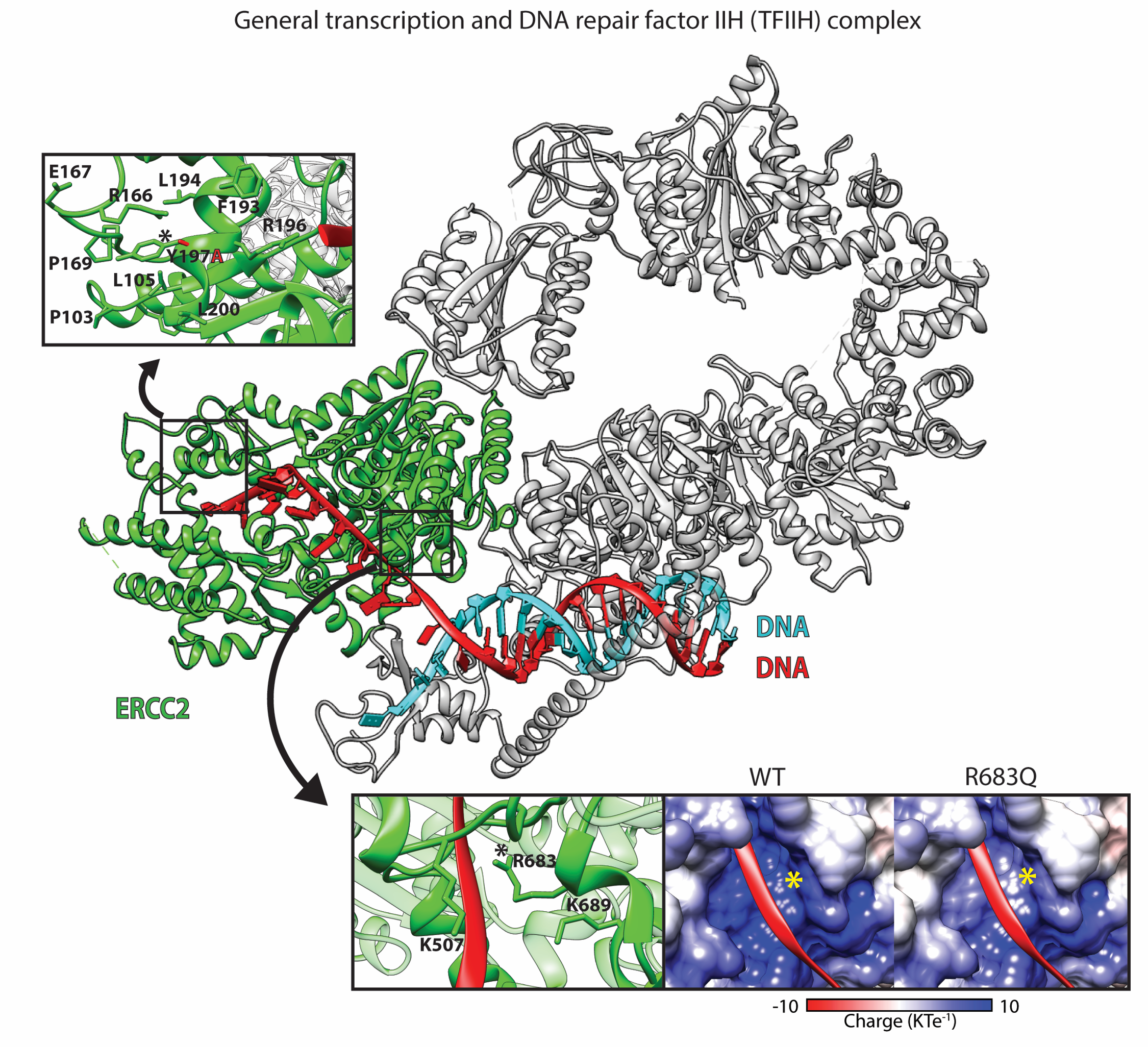

Supplement: Supplementary file 5 — Additional file 5. Figure S4. Structural basis for TTD mutations in trichothiodystrophy. [file 40246_2024_603_MOESM5_ESM.tif]

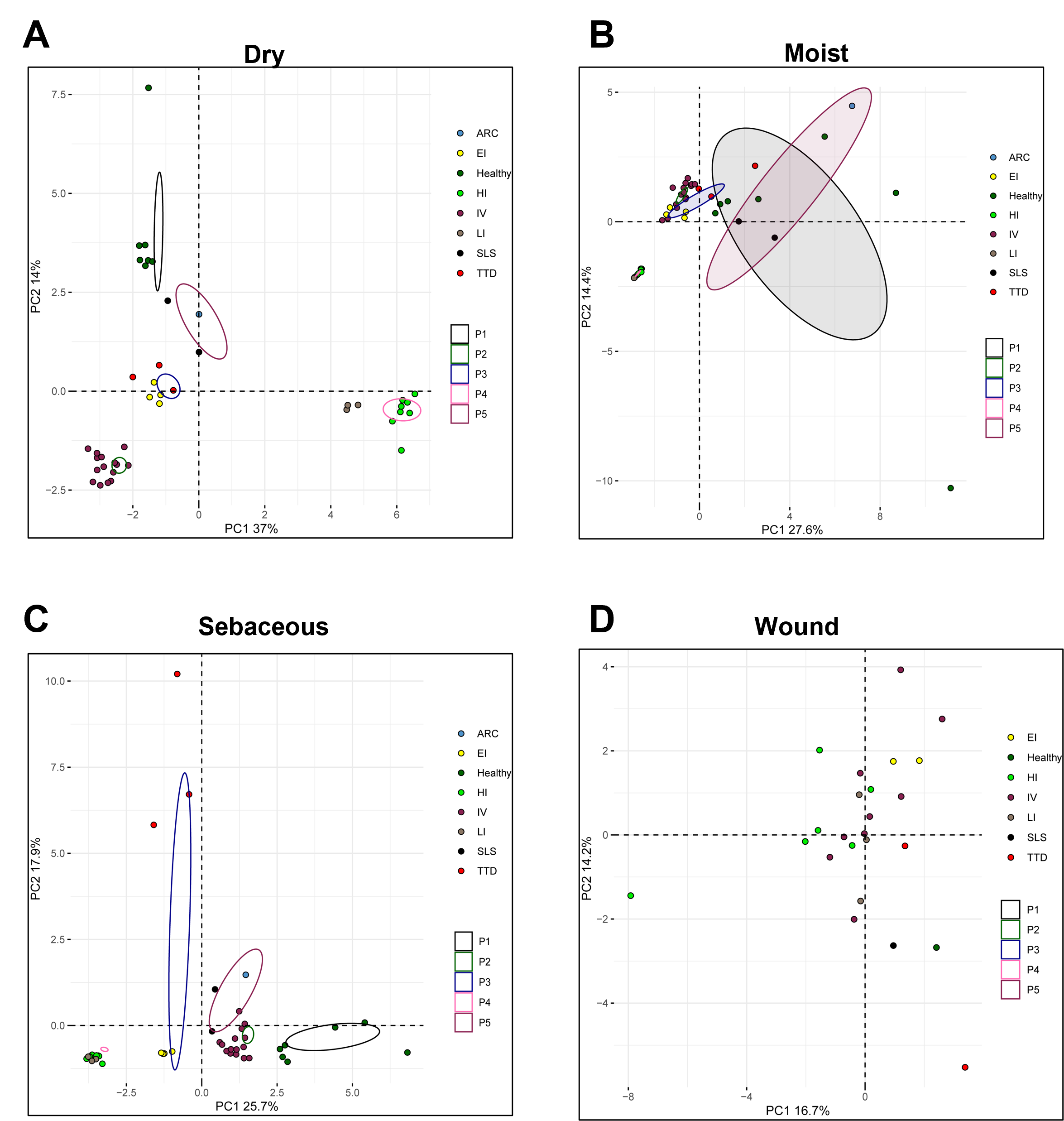

Supplement: Supplementary file 6 — Additional file 6. Figure S5. PCA analysis for grouping. [file 40246_2024_603_MOESM6_ESM.tif]

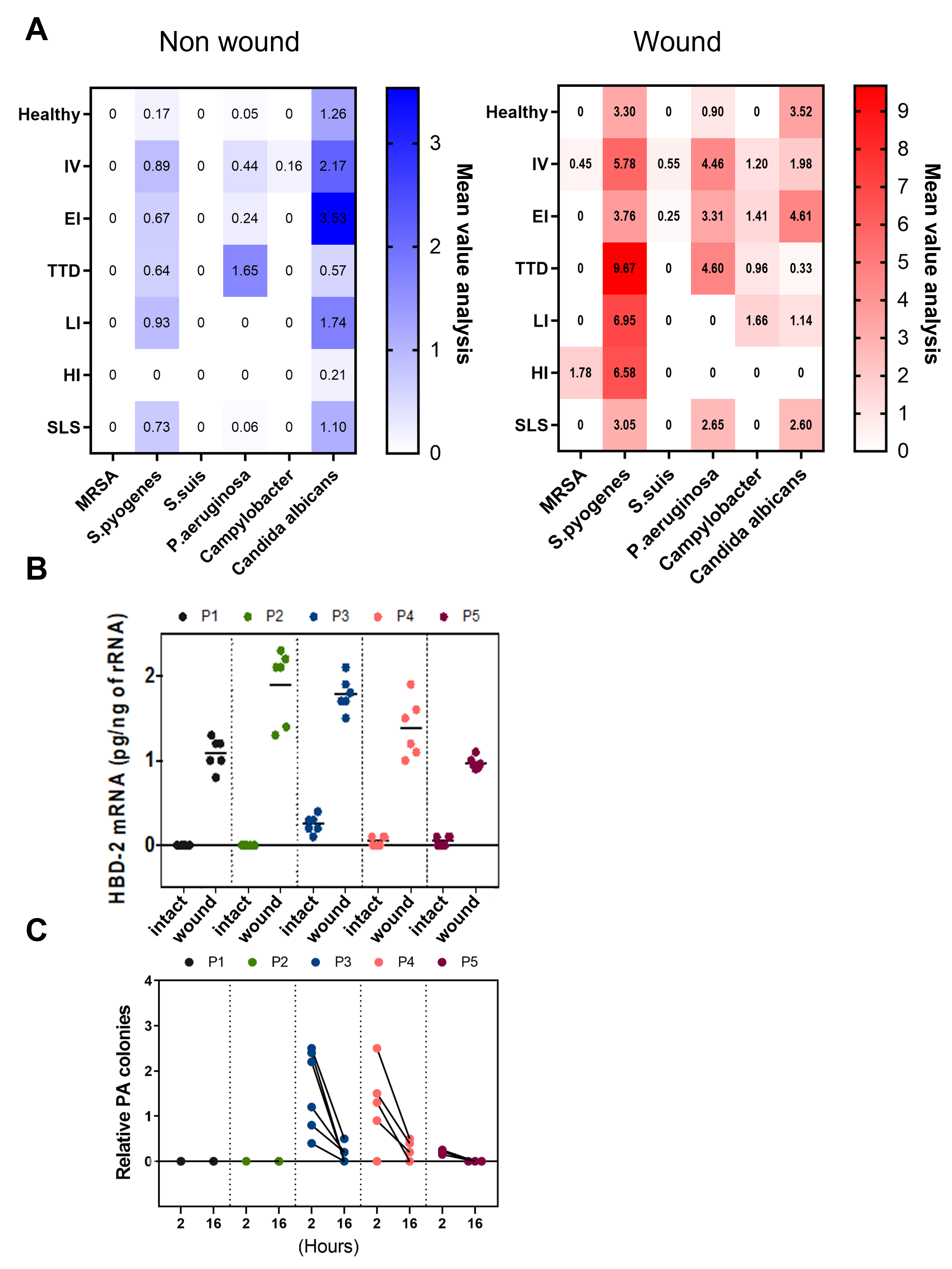

Supplement: Supplementary file 7 — Additional file 7. Figure S6. Microbiota and antimicrobial peptide changes in wounded CI skin compared to nonwounded skin. [file 40246_2024_603_MOESM7_ESM.tif]

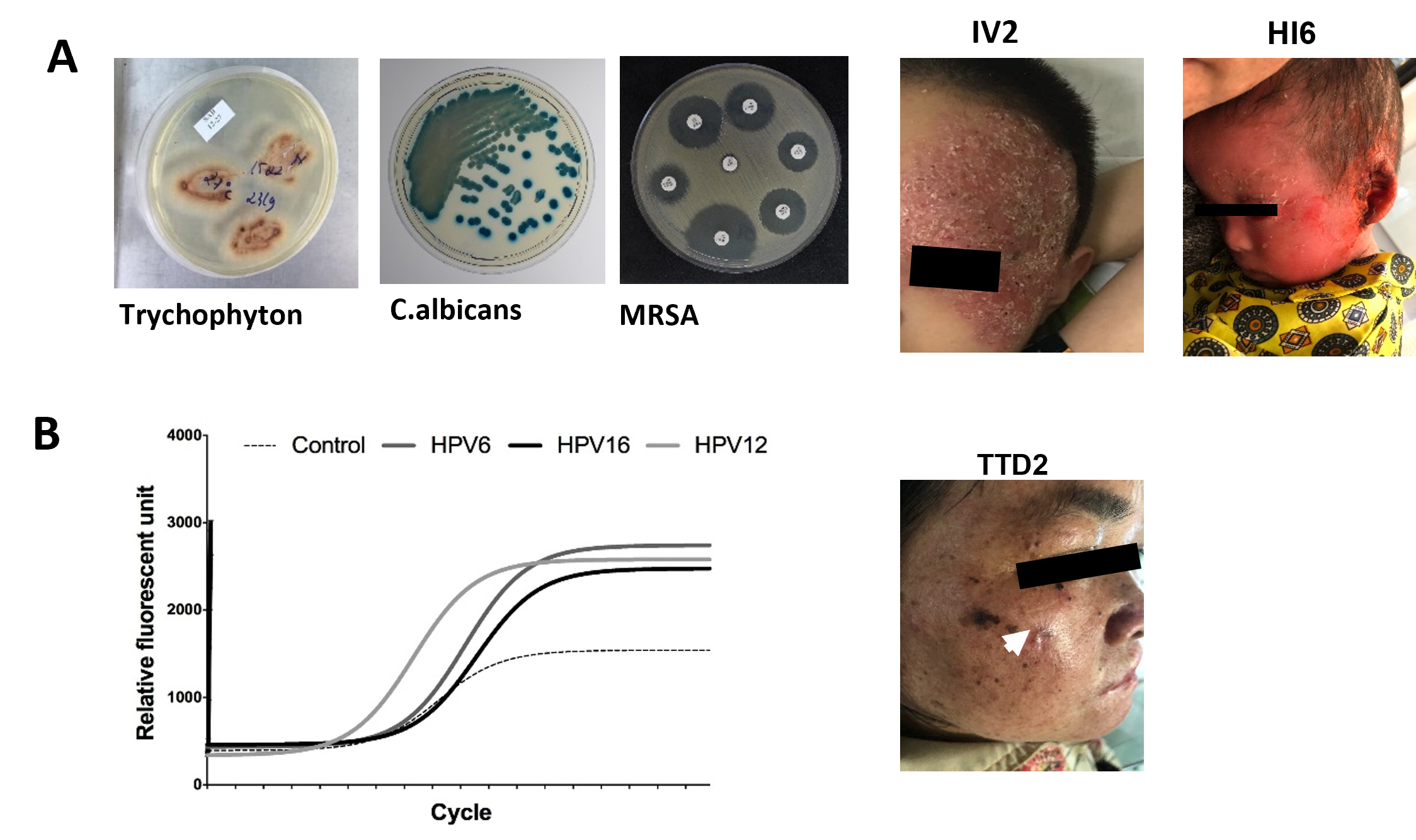

Supplement: Supplementary file 8 — Additional file 8. Figure S7. Presence of microbial and viral species in CI patients. [file 40246_2024_603_MOESM8_ESM.tif]

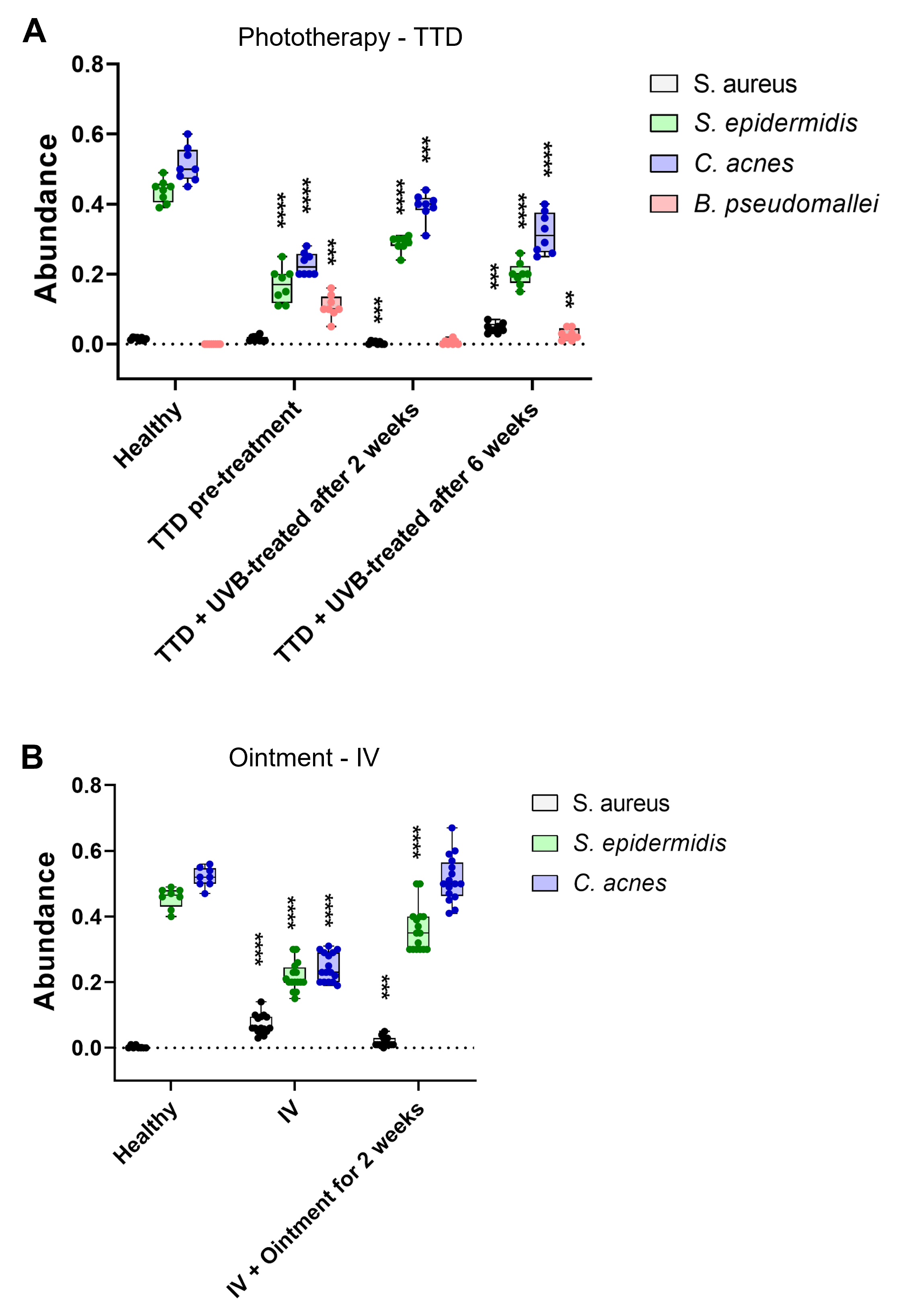

Supplement: Supplementary file 9 — Additional file 9. Figure S8. Restoration of CI patient microbiome homeostasis with treatments of TTD and IV. [file 40246_2024_603_MOESM9_ESM.tif]

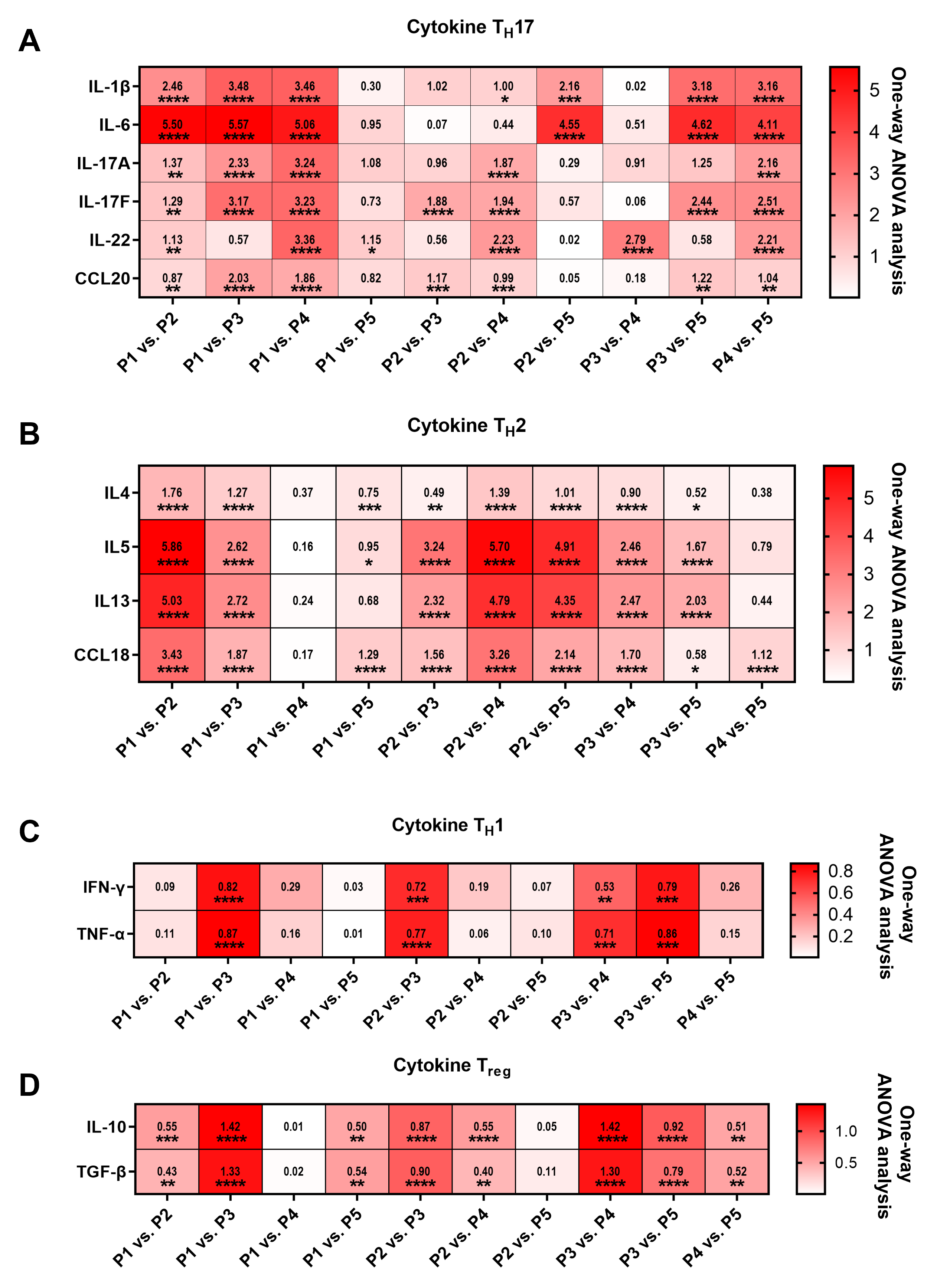

Supplement: Supplementary file 10 — Additional file 10. Figure S9. One-way ANOVA analysis of cytokine expression levels across the 5 CI dysbiosis clusters. [file 40246_2024_603_MOESM10_ESM.tif]

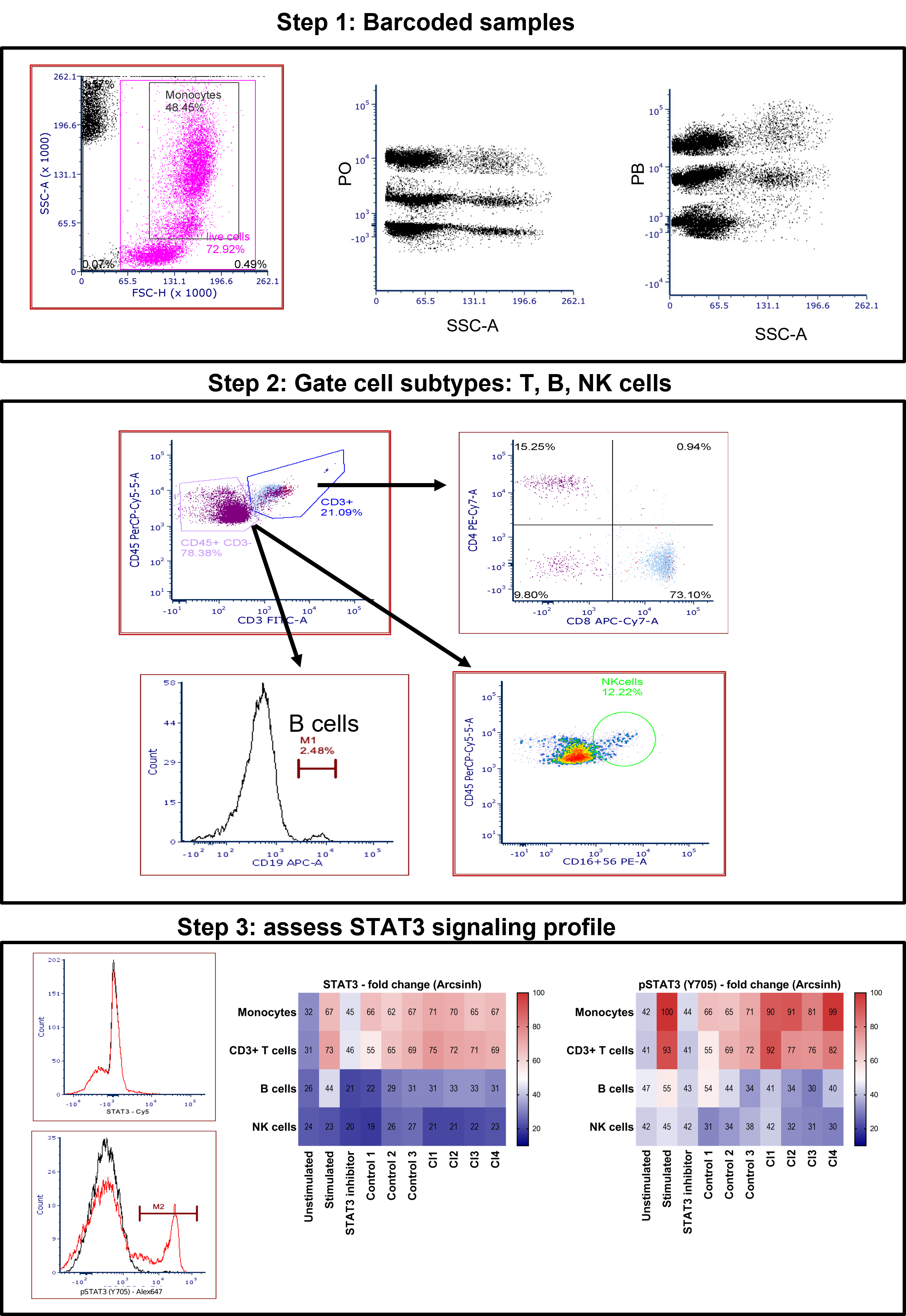

Supplement: Supplementary file 11 — Additional file 11. Figure S10. Procedure for determining JAK/STAT signaling profile in CI patients. [file 40246_2024_603_MOESM11_ESM.tif]

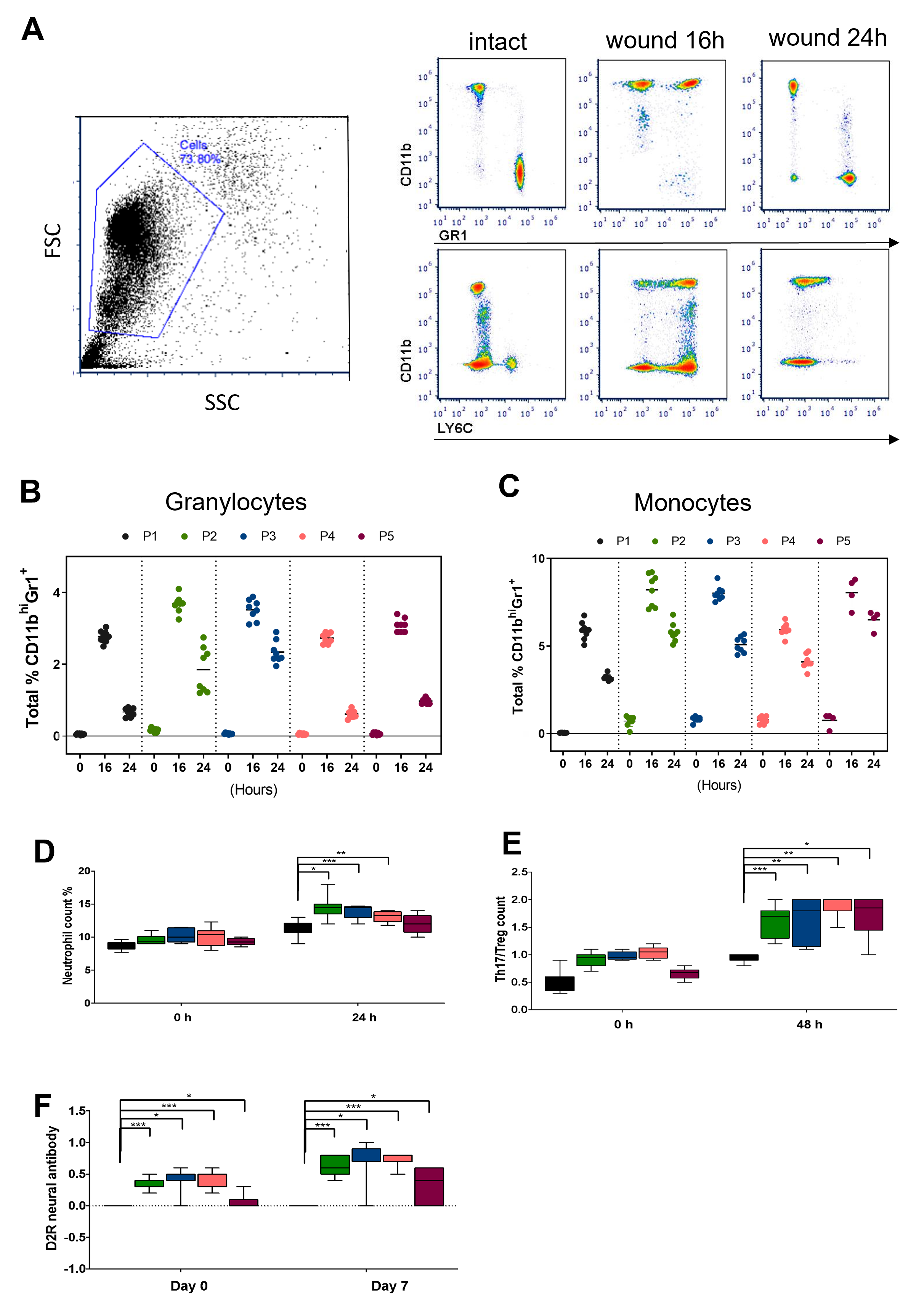

Supplement: Supplementary file 12 — Additional file 12. Figure S11. Relative granulocyte and monocyte inflammation in CI patients following wounding of skin. [file 40246_2024_603_MOESM12_ESM.tif]

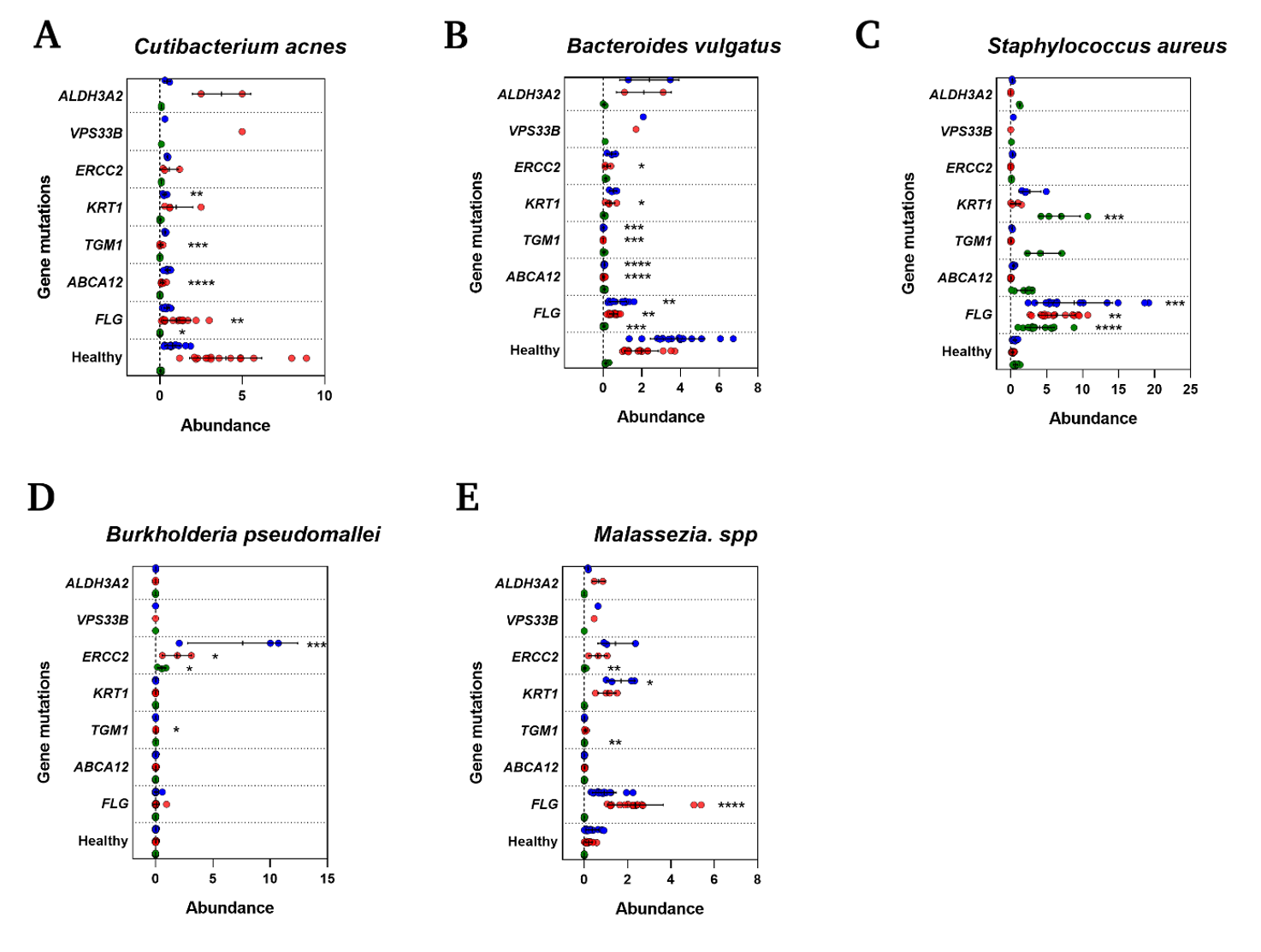

Supplement: Supplementary file 13 — Additional file 13. Figure S12. Bacterium abundance plotted against CI gene mutation. [file 40246_2024_603_MOESM13_ESM.tif]
